# Hydrochemical gradients driving extremophile distribution in saline and brine groundwater of southern Poland

**DOI:** 10.1111/1758-2229.70030

**Published:** 2024-10-23

**Authors:** Mirosław Słowakiewicz, Weronika Goraj, Tomasz Segit, Katarzyna Wątor, Dariusz Dobrzyński

**Affiliations:** ^1^ Faculty of Geology University of Warsaw Warsaw Poland; ^2^ Faculty of Medicine The John Paul II Catholic University of Lublin Lublin Poland; ^3^ Faculty of Geology, Geophysics and Environmental Protection AGH University of Krakow Kraków Poland

**Keywords:** brine, extremophiles, halophiles, hydrochemical gradients, methanogens, saline groundwater, sulphate metabolism

## Abstract

Extreme environments, such as highly saline ecosystems, are characterised by a limited presence of microbial communities capable of tolerating and thriving under these conditions. To better understand the limits of life and its chemical and microbiological drivers, highly saline and brine groundwaters of Na‐Cl and Na‐Ca‐Cl types with notably diverse SO_4_ contents were sampled in water intakes and springs from sedimentary aquifers located in the Outer Carpathians and the Carpathian Foredeep basin and its basement in Poland. Chemical and microbiological methods were used to identify the composition of groundwaters, determine microbial diversity, and indicate processes controlling their distribution using multivariate statistical analyses. DNA sequencing targeting V3‐V4 and V4‐V5 gene regions revealed a predominance of Proteobacteriota, Methanobacteria, Methanomicrobia, and Nanoarchaea in most of the water samples, irrespective of their geological context. Despite the sample‐size constraint, redundancy analysis employing a compositional approach to hydrochemical predictors identified Cl/SO_4_ and Cl/HCO_3_ ratios, and specific electrical conductivity, as key gradients shaping microbial communities, depending on the analysed gene regions. Analysis of functional groups revealed that methanogenesis, sulphate oxidation and reduction, and the nitrogen cycle define and distinguish the halotolerant communities in the samples. These communities are characterised by an inverse relationship between methanogens and sulphur‐cycling microorganisms.

## Graphical abstract

The solute composition differentiates high‐salinity groundwaters of southern Poland into three genetic, aquifer‐related groups. Sulphate and hydrogen carbonate concentrations relative to chloride, along with total salinity, are the key factors controlling microbial communities. Methanogens dominate in sulphate‐poor brines of the Carpathian Foredeep and flysch formations, while sulphur‐cycling microorganisms, except for thiosulphate oxidisers, are abundant in high‐sulphate waters of the Carpathian Foredeep basement. Total salinity regulates halophiles, and more intricate mechanisms govern methanotroph distribution.

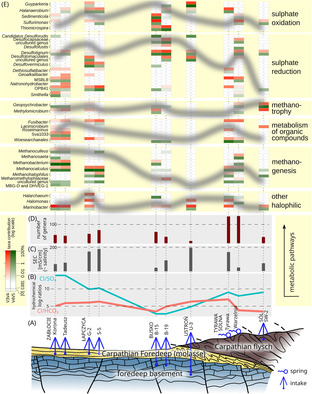

## INTRODUCTION

Saline waters and brines are commonly occurring groundwater types in the stagnant zone of hydrogeological systems and in some locations at surface water reservoirs. The distinction between saline water and brine is related to their mineralisation (total dissolved solids [TDS]), and according to the most commonly used USGS classification (Heath, 1983), saline waters have TDS between 1 and 35 g/L, whereas brines have TDS >35 g/L. Subsurface and surface hypersaline environments are preferred habitats of a wide variety of microorganisms which can withstand osmotic pressure and chaotropic stress, thus expanding the habitability on Earth beyond aqueous environments. These settings, in particular in brines, have become astrobiologically important due to the recent detection of salts and the hypothetical presence of water reservoirs on Jupiter, Europa, and Mars (Martínez & Renno, 2013; McKinnon & Zolensky, 2003). Understanding life in extreme salty ecosystems on Earth can expand our knowledge about the limitations of life and bring an opportunity to reconstruct life conditions on the early Earth and other planets.

Current research on microbial communities capable of tolerating (halotolerant) or thriving (halophilic) in high salt concentrations primarily focuses on the pollutant‐degrading pathways these microorganisms employ in high salinity groundwater (Wang et al., 2024). Halophilic and halotolerant communities have evolved specialised genes, physiological mechanisms and metabolites that offer significant potential for biotechnological applications derived from saline environments (J. Wang, Liu, et al., 2023). A novel application of these extremophiles involves hydrogen storage in salt caverns, where microorganisms are in direct contact with the stored hydrogen for extended periods. This interaction induces various processes, such as sulphate reduction, which produces toxic H_2_S, or methanogenesis by archaea, leading to the formation of CH_4_, which can diminish the energetic value of the stored gas (Dopffel et al., 2023). Additionally, isotopic tracing methods using stable isotopes like ^2^H, ^18^O, ^14^C, and strontium isotopes have provided valuable insights into the sources, ages, and recharge rates of saline and brine groundwaters (Benetti et al., 2017; Kani et al., 2023; Xu et al., 2023). Beyond environmental, biotechnological, industrial, or agricultural perspectives, water‐bearing rocks, in some cases including evaporites, are very diverse in terms of their chemical and mineral composition. This diversity leads to groundwater with varied chemical profiles, particularly in terms of minor and trace elements, which likely influence the composition and activity of microbial communities (Payler et al., 2019). Despite growing interest, the literature on bacteria and archaea in subsurface saline waters and brines remains limited. This knowledge gap extends to methanogenesis and its relation to the carbon cycle in these extreme environments. Given the environmental aspect, salinity has been suggested to affect CH_4_ emission rates from terrestrial strata (Qu et al., 2024), to increase CH_4_ and N_2_O concentrations as TDS decrease (Li et al., 2021), to significantly shape the spatial distribution of prokaryotic communities (Liu et al., 2022), and to generate CH_4_ in hypersaline lakes (Kallistova et al., 2020).

To further examine the impact of high‐TDS groundwater on deep subsurface microorganisms, samples were collected from relic saline waters and brines in the Carpathian Mountains and Carpathian Foredeep basin, in southern Poland. The term “relic” is used here in the broad sense meaning high TDS groundwater whose chemical composition has resulted from geochemical processes operating in the geological past, as opposed to saline waters and brines whose composition results from modern leaching of salt‐bearing formations. This study also aims to determine the key parameters that control microbial distribution and functional capabilities of bacteria and archaea. To this end, the following hypothesis was tested: the chemical composition of saline and brine groundwater is related to the taxonomic composition and functional roles of the microbial community that shape the microbiota. In addition, because the studied groundwater contains CH_4_, special attention was also paid to methanogenic versus methanotrophic consortia in order to find factors controlling their distribution.

## EXPERIMENTAL PROCEDURES

### Sampling and physicochemical analyses of groundwater

The following main criteria guided the selection of groundwater for testing: (i) examining waters with the highest mineralisation in the region and (ii) selecting waters representing different geological histories and geological environments (different chemical and mineral composition of aquifer rocks).

Ten groundwater samples have been collected from eight deep water wells (in Busko: B‐15, B‐19; Łapczyca: G‐2, S‐5; Sól: SW‐2; Ustroń: U‐3; Zabłocie: Korona; Tadeusz) and two springs (Tyrawa and Warzelnia in Tyrawa Solna) (for geological and hydrogeological outlines see supporting information and Table S1) into sterile 1 L glass bottles (2 L of water/sample). All samples were immediately sent for chemical analyses.

Water samples for metal analyses were filtered in the field by nylon membrane filters of 0.45 μm pore size (Whatman, USA), preserved with ultra‐pure nitric acid (Ultrex, JT Baker, USA) and stored in LDPE bottles (Nalgene, USA). Physicochemical parameters (pH, E_H_, T, and specific electrical conductivity [SEC]) were measured in the field by using a CX‐461 multifunctional instrument (Elmetron, Poland).

Chemical analyses of groundwater samples were performed in the accredited Hydrogeochemical Laboratory of the AGH University of Krakow (see supporting information for technical details).

### DNA extraction, amplification, and sequencing

Water samples (1000 mL) were filtered through sterile polycarbonate membranes with 0.22 μm pore size (EMD Millipore, USA). Filters were fragmented into pieces, put into the bead‐beating solution of the DNeasy PowerMax Soil Kit (Qiagen, USA) and processed according to the manufacturer's protocol. Microbial DNA‐free water (Thermo Fisher Scientific, USA) was filtered and used for DNA extraction, the subsequent amplification, and sequencing as a negative‐control sample. DNA concentration was measured using Qubit 2.0 (Invitrogen, USA) and Qubit dsDNA HS Assay Kit (Thermo Fisher Scientific).

The amplicons were prepared using bacterial (341F‐CCT ACG GGN GGC WGC AG and 785R‐GAC TAC HVG GGT ATC TAA TCC) (Klindworth et al., 2013) and archaeal (513F‐GGT GYC AGC CGC CGC GGT AA; 915R‐GTG CTC CCCCGC CAA TTY CT) (Deja‐Sikora et al., 2019) primers targeting the V3‐V4 and V4‐V5 16S rRNA gene region, respectively. Each sample was amplified with NEBNext® High‐Fidelity 2 × PCR Master Mix (New England BioLabs) according to the manufacturer's instructions. Paired‐end (2 × 250 nt) sequencing was performed with an Illumina MiSeq by Genomed S.A. (Warsaw, Poland) and following the manufacturer's run protocols (Illumina, Inc., San Diego, CA, USA).

### Sequence data pre‐processing and taxonomic assignment

Automatic preliminary data processing was performed on the MiSeq using MiSeq Reporter v2.6 software. The analysis consisted of two steps: automatic demultiplexing of samples and generation of FASTQ files containing raw reads. Bioinformatic analysis ensuring the classification of the readings to the genus level was carried out with the QIIME software package (Caporaso et al., 2010) based on the SILVA_v_138 reference sequence database (Quast et al., 2013). The analysis consisted of the following steps: (i) removal of adaptor sequences using Cutadapt (Martin, 2011); (ii) analysis of read quality and removal of low‐quality sequences (quality <20, minimum length 30) using Cutadapt; (iii) joining of paired sequences using fastq‐join (16S) algorithm; (iv) sequence chimera removal using the usearch61 algorithm (Edgar, 2010); (v) clustering based on a selected reference sequence database using the uclust algorithm (Edgar, 2010); and (vi) assigning taxonomies to a selected reference sequence database using the uclust algorithms (Altschul et al., 1990). A simple yet effective strategy to remove potentially spurious OTUs was applied, using a cutoff of 0.25% relative abundance in at least one sample (Reitmeier et al., 2021). Since most OTUs were represented by very low abundances, this approach reduced their number by 83 and 85.5%, while decreasing the total number of reads by only 7.5 and 9% for the V3‐V4 and V4‐V5 amplified data, respectively.

### Analysis of hydrochemical and sequencing data

The physicochemical properties and composition of the groundwater samples were analysed using correlation coefficients, compositional variation and agglomerative clustering. Microbial community data were converted to distance representations. Then, associations between the hydrochemical and microbial datasets were explored through distance‐based redundancy analysis (db‐RDA). The detailed protocol for the statistical methods used is explained in supporting information.

## RESULTS

### Physicochemical characteristics of groundwater

**FIGURE 1:**
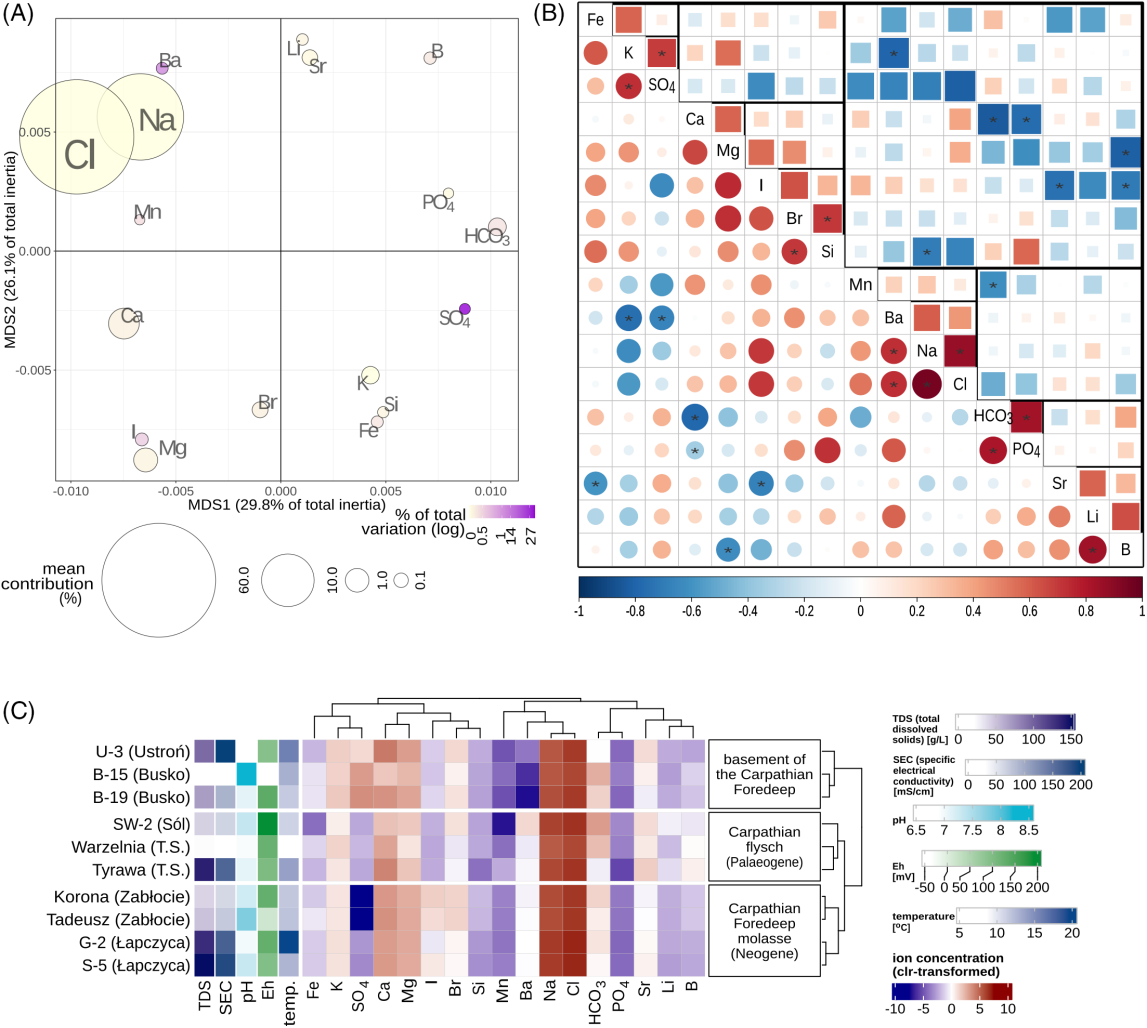
Exploring relationships of hydrochemical components of groundwater samples. (A) Mapping of weighted correlation distances between ions using MDS, complemented by the representation of mean ion contribution and compositional variation. (B) Correlogram of ions (Spearman's correlation; upper triangular matrix‐weighted coefficients, lower—unweighted), grouped by hierarchical clustering with average linking method; branches of a triangular dendrogram indicated by thickness of the grid lines; “*”—coefficient significant at *p* = 0.05. (C) Ion composition and hydrochemical parameters; data are arranged based on hierarchical relationships among ions and calculated on weighted coordinates.

Regarding the major solute concentrations, the chemical composition of the studied groundwaters is dominated by chloride and sodium, typical of the Na‐Cl hydrochemical type of water (Figure 1A, supporting information Table S1). Only one water (U‐3 from Ustroń) has an elevated relative proportion of calcium (Na‐Ca‐Cl hydrochemical type). The groundwaters studied are characterised by TDS between 20.5 and 151.1 g/L, pH ranging between 6.9 and 8.2, and near neutral redox potential (E_H_) from −37 to 193 mV (Figure 1C, supporting information Table S1). Sulphate ions usually occur in low concentrations (less than 11 mg/L) and were not detected in Zabłocie (concentration below limit of quantification); only waters from Busko and Ustroń have significantly elevated SO_4_ concentrations, that is, 750–2680 and 350 mg/L, respectively.

The analysed brines and saline waters are usually enriched in elements characteristic of seawater, such as Li, Br, I, and B. In addition, despite differences in genesis of the groundwaters studied, these elements are present in markedly elevated although varying concentrations, for example, iodine (concentrations ranging from 3 to 198 mg/L) or boron (8 to 205 mg/L) (Supporting information Table S1).

The evaluation of relationships between variables reveals strong, usually statistically significant, positive pairwise correlations Na/Cl, HCO_3_/PO_4_, K/SO_4_, Br/Si, and negative correlations SO_4_/Mn (Ba, Na, Cl), Ca‐HCO_3_ (PO_4_), K/Ba, I/Sr (Li, B) (Figure 1B). Some negative co‐associations are likely controlled by precipitation of mineral phases (e.g., baryte, carbonates, phosphates) or complexing, but other relations may have a complex source, associated with the origin of groundwaters, including the effects of water–rock interactions, diagenetic processes, ultrafiltration, and mixing of various waters. The variability between solutes, as expressed by the total compositional variation (Aitchison, 1982), is mostly shared by SO_4_ and Ba, followed by I, Mn, and HCO_3_ of much lower, but still elevated contributions to the variation (Figure 1A).

The clustering of cases (the studied water samples) perfectly reflects the three general types of geological environments in which these waters occur, that is, (a) waters of Tyrawa Solna and Sól in flysch rocks of the Outer Carpathians, (b) waters of Łapczyca and Zabłocie in the Miocene deposits of the Carpathian Foredeep basin, and (c) waters of Busko and Ustroń in rocks forming the basement of the Carpathian Foredeep basin (Supporting information Figure S1, Table S1). The components that contribute much to the distances between the three aforementioned clusters include SO_4_, Ba, K, Sr‐Li‐B, and I. The groundwaters within cluster (iii) constitute the most distinct group among the samples, primarily marked by highly elevated sulphate levels that are positively correlated with K and negatively correlated with Ba. The cluster (i) differs from (ii) with the higher Sr, Li, and B concentration (forming a distinct variable cluster) and significantly lower I along with slightly lower SO_4_ concentration (Figure 1C).

### Composition of microbial communities

The percentages of archaeal and bacterial sequences identified using primers targeting the V3‐V4 and V4‐V5 16S rRNA gene region are diversified. When universal bacterial/archaeal primers were used, the relative abundance of archaea ranged from 0 to ~3%, whereas the use of primers specifically targeting the archaeal 16S ribosomal RNA gene region resulted in abundance of archaea ranging from 0 to 86% (Supporting information Figure S2).

**FIGURE 2:**
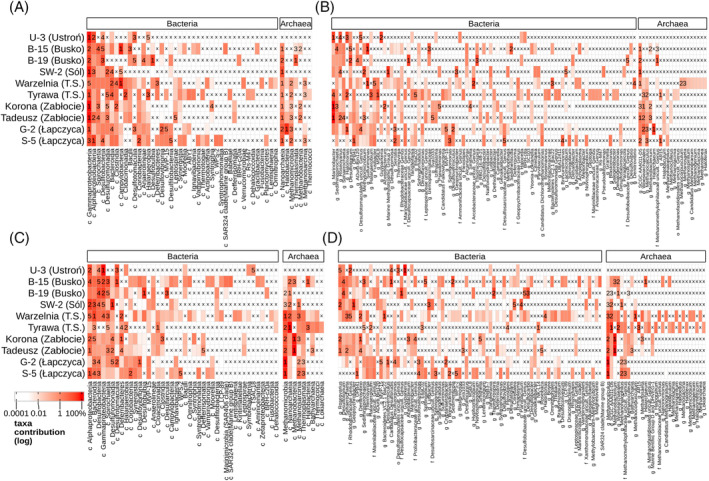
Taxonomic composition of microbial communities as assessed by 16S rRNA gene amplicon sequencing. Gene regions data for (A) and (B): V3‐V4, for C‐D: V4‐V5; (A) and (C) classes, (B) and (D) genera (bacteria—only the most abundant 68% of taxa); abundance calculated with values imputed for zeros; prefixes other than “c” (class), and “g” (genus) indicate sequences resolved at higher than nominal taxonomic levels (f‐family, o‐order); ‘x”—taxon absent.

The Proteobacteria phylum is represented by the Gammaproteobacteria and Alphaproteobacteria classes, with Gammaproteobacteria commonly being the predominant component (Figure 2A,C). For example, in the Korona and Tadeusz water samples, it comprises 80% or more of the total microbial community. However, other waters demonstrate higher diversity at the class level. In addition to Gammaproteobacteria and Alphaproteobacteria, the classes such as Bacilli (~9% in B‐15), Bacteroidia (~11% in Warzelnia), Campylobacteria (~56% in B‐15 and 63% in Warzelnia), Cloacimonadia (~23% in Tyrawa and ~ 16% in S‐5), Desulfobacteria (~23% in B‐19 and 5% in S‐5), Desulfobulbia (~29% in B‐19), Desulfuromonadia (~9% in SW‐2), Halanaerobiia (15% in Tyrawa), Halobacteria (~46% in G‐2), Nanoarchaeia (~15% in W‐2 and ~8% in G‐2), and Spirochaetia (~8% in B‐15) have been identified.

Analysis of the V4‐V5 region enabled a more precise examination of the microbial community composition, focusing on archaea, primarily methanogens (Figure 2C). Both samples from Zabłocie indicate the presence of the Methanobacteria (65–77%) and Methanomicrobia (11–13%), whereas in Łapczyca only Methanomicrobia (59–76%) is observed. In the Warzelnia saline water sample, the classes Methanomicrobia (~30%), Nanoarchaeia (~9%), and Methanosarcinia (~5%) are present, whereas in Tyrawa brine, Nanoarchaeia (74%) and Methanomicrobia (~13%) were found. In SW‐2 brine, archaea make up a negligible part of the microbiome and in U‐3 brine, no sequences classified as archaea were identified (Figure 2D).

Analysis of the core microbiome at the genus level (top 5 taxa) indicates the presence of bacteria belonging to three groups, that is, sulphur bacteria, bacteria that oxidise or reduce iron and manganese, and methanotrophs (Figure 2A,B). Furthermore, amplification of the V4‐V5 region also increased the relative abundance of archaeal genera associated with methanogens (Figure 2C,D). *Marinobacter* dominates in both Zabłocie and U‐3 samples, with a prevalence exceeding 30% (Figure 2B). This type of bacteria represents aerobic chemoheterotrophs but can also grow anaerobically through denitrification coupled with the oxidation of the corresponding donor of the carbon substrate. They are also halotolerant and grow in 4.7–205 g/L NaCl. However, distinct samples from the same locations manifest variations in microbiome composition (Figure 2A–D). Significant differences are observed in the types of groundwater in Busko. In B‐15 saline water, the dominant genera are *Sulfurimonas* (~56%), *Sedimenticola* (~9%), and *Desulfotignum* (4%), whereas in B‐19 brine uncultured Desulfocapsaceae (~27%), *Desulfotignum* (~23%), and *Thiomicrospira* (~21%) prevail. The Tyrawa Solna samples are also quite diverse. The Warzelnia saline water is characterised by the dominance of *Sulfurimonas* (26%), uncultured Arcobacteraceae (~21%) and Bacteroidetes_VC2.1_Bac22 (~10%). *Sulfurimonas* and *Thiomicrospira* are chemolithotrophic or chemolithoautotrophic bacteria for which the electron donor is reduced sulphur compounds and the electron acceptor is oxygen (under aerobic conditions) or nitrate (under anaerobic conditions). *Sedimenticola* and *Desulfotignum* are types of chemoorganotrophic bacteria that use organic compounds (e.g., organic acids, alcohols). *Sedimenticola* can use a variety of electron acceptors, including oxygen (under aerobic conditions) and sulphate (under anaerobic conditions), whereas *Desulfotignum* is a strictly anaerobic bacterium that mainly uses sulphate. Both samples of Tyrawa Solna consist mainly of MSBL8 (Cloacimonadales) (~23%), *Halomonas* (~20%), and *Halanaerobium* (~15%). The *Halomonas* bacteria exhibit metabolic versatility, enabling them to thrive in both aerobic and anaerobic environments. They employ diverse metabolic pathways, including hydrocarbon degradation, sulphur oxidation and denitrification. In contrast, *Halanaerobium* is strictly anaerobic, primarily engaging in fermentative processes and sulphate reduction. Interestingly, Marine Methylotrophic Group 2 (~41%), that is, methylotrophs belonging to the order Methylococcales, and methanotrophs of the genus *Methylomicrobium* (~19%), are abundant only in SW‐2 brine (Figure 2B). With regard to archaea, important genera include *Methanobacterium*, *Methanocalculus*, and *Methanohalophilus* methanogens, present in a majority of samples, where they account for 1–89%. In contrast, no methanogens were found in the U‐3 brine sample.

### Relationships of hydrochemical data with the microbial community composition

**FIGURE 3:**
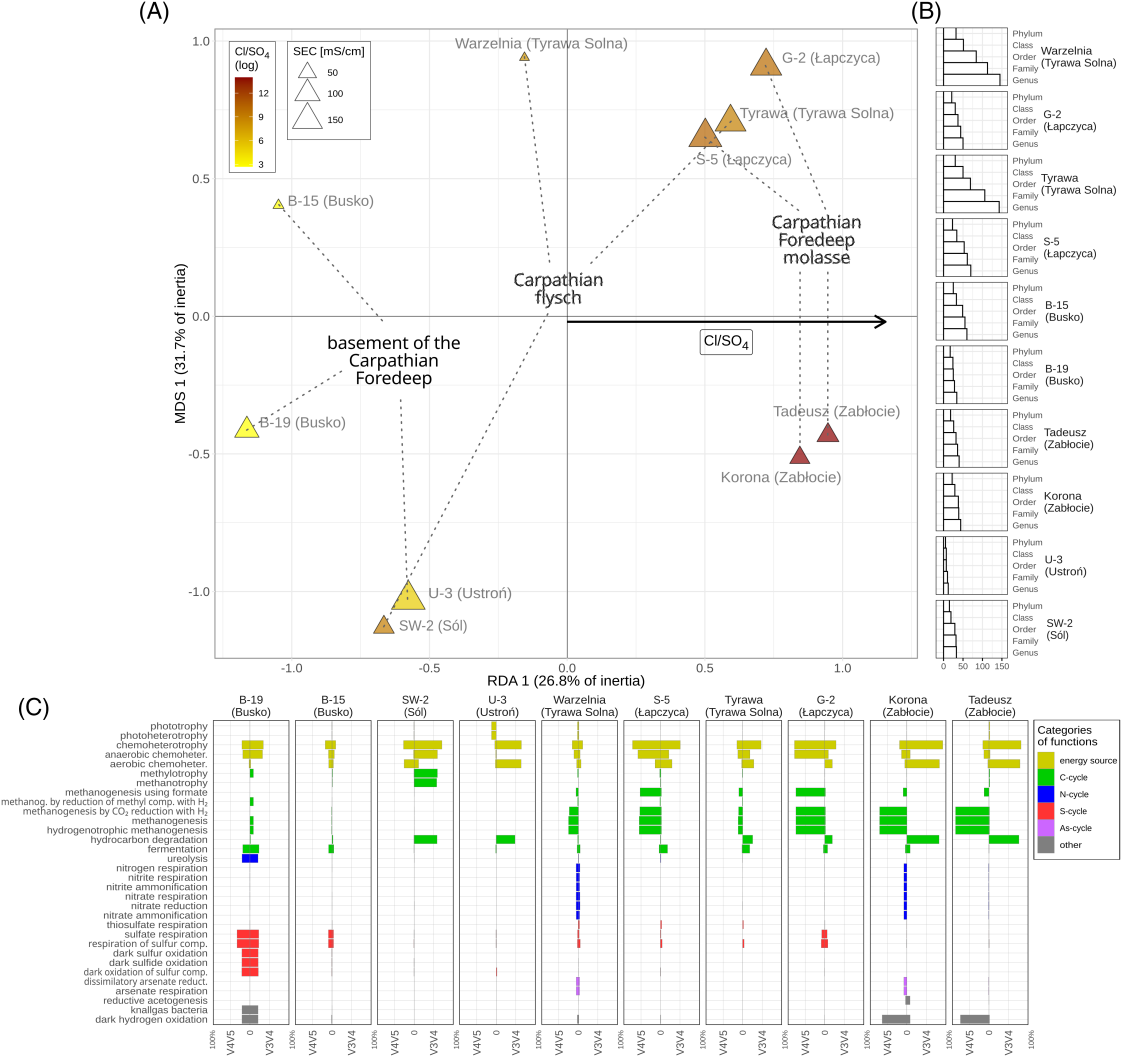
(A) db‐RDA biplot based on 16S rRNA amplicon sequence data, combined gene regions V3‐V4 (bacteria) and V4‐V5 (archaea), WGSUniFrac distance; this ordination illustrates the relationship between ion log‐ratio that best explain variation of microbial assemblages along constrained RDA 1 axis and variation distributed along unconstrained MDS 1 axis; site scores are annotated with groundwater aquifers; SEC‐specific electrical conductivity. (B) Taxa richness at different taxonomic ranks, reflecting diversity gradient along MDS 1 axis and (C) percentage of OTUs (>2%; weighted by abundance data) with specific trait in communities, as inferred from FAPROTAX database (Louca et al., 2016) using unfiltered sequence data; separate bars for amplified V3‐V4 and V4‐V5 gene regions, distribution of facets corresponds to the order of site scores along RDA 1.

**FIGURE 4:**
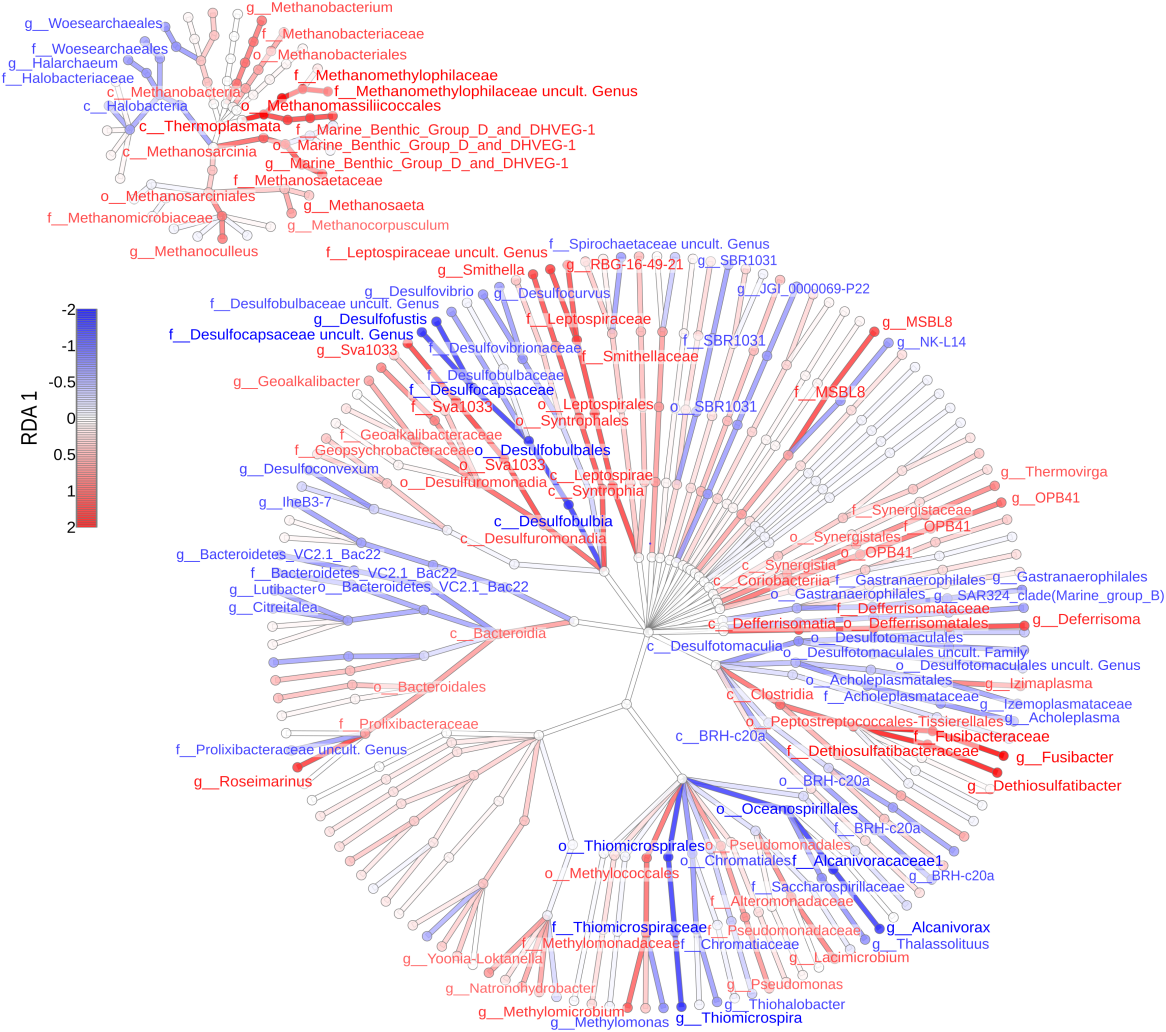
Overall taxonomic structure of the analysed groundwater microbial communities at the genus to class levels. Node's colour intensity corresponds to taxa score on the positive (red shades) and negative (blue shades) half‐axes of the RDA 1 axis (WGSUniFrac distance, combined bacterial and archaeal sequences data derived from the V3V4 and V4V5 16S rRNA sectors, respectively); taxon names represent top ~30% most contributing scores to the axis at each taxonomic level.

**FIGURE 5:**
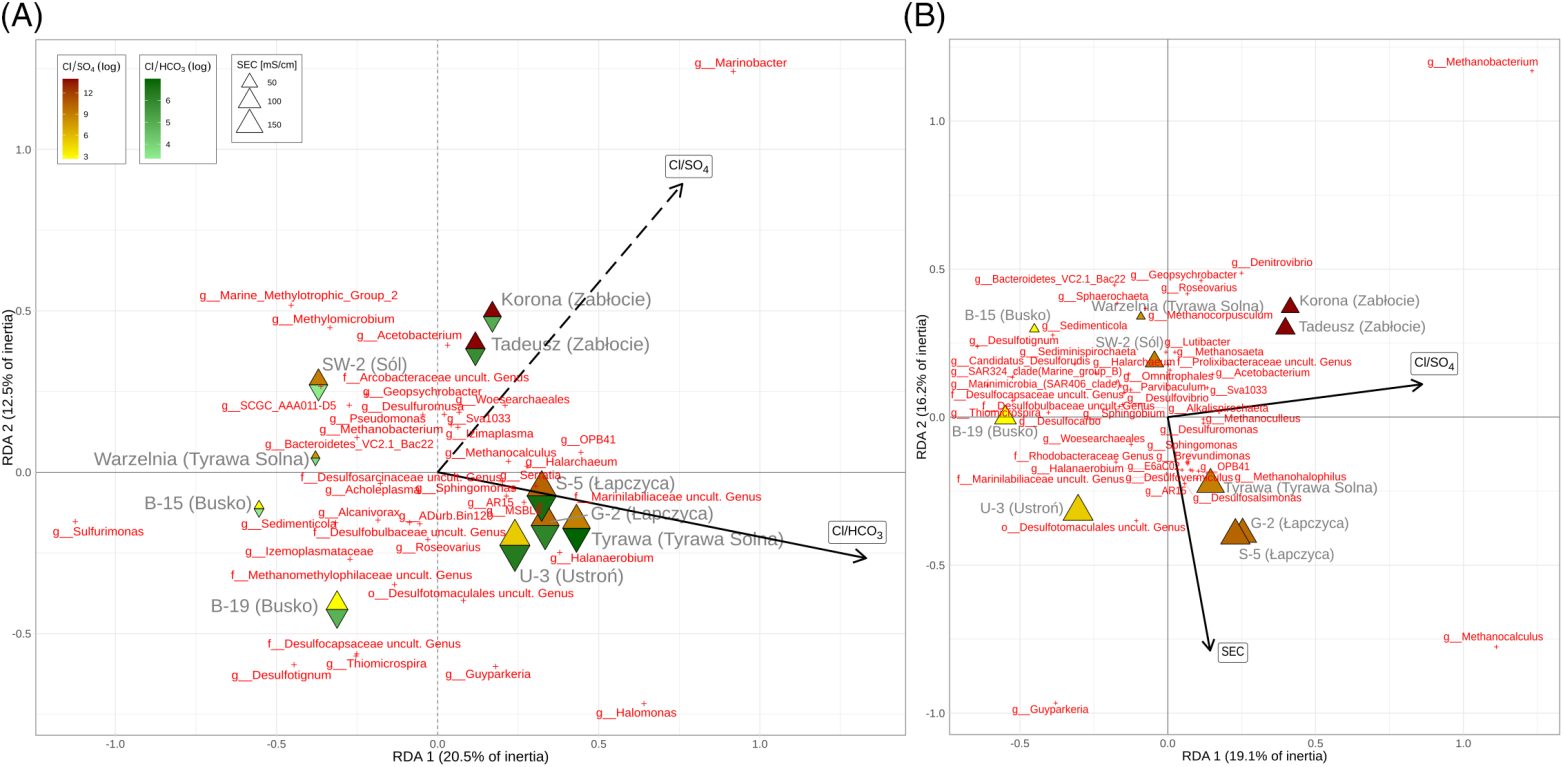
db‐RDA ordination triplot showing site scores, loadings and Hellinger‐transformed genera scores added to the ordination. (A) V3‐V4 gene region data and (B) V4‐V5 gene region data; Bray–Curtis dissimilarity; dashed lines—axes and loadings non‐significant at the p = 0.05 level, SEC—specific electrical conductivity.

The Cl/SO_4_, Na/Ca, Mg/HCO_3_, Cl/HCO_3_, and K/Ba variables collectively accumulated >99% of the log‐ratio variation. The global db‐RDA models, created using Bray‐Curtis and WGSUniFrac distances, were statistically significant. The significant predictors and the number of constrained axes that remained in the final models after variable selection varied between the models. In the model constructed on the basis of WGSUniFrac distance, only one predictor (Cl/SO_4_) and consequently one axis represented the constrained part of ordination, accounting for ~27% of the variation. Notably, the first unconstrained axis contributed even more to the total variance, at ~32% (Figure 3A). The classes associated with the largest positive scores on the RDA1 axis are Clostridia, Coriobacteria, Deferrisomatia Syntrophia, Leptospirae, and Methylococcales among the bacteria, and Methanosarcinia and Thermoplasmata among the archaea. The classes Desulphobulbia, Thiomicrospirales, Oceanospirillales, and Halobacteria (archaea) show the most negative scores (Figure 4). The genera with the highest scores, mostly but not exclusively belonging to these classes, include bacteria such as *Dethiosulfatibacter*, *Fusibacter*, *Deferrisoma*, OPB41, *Smithella*, *Geoalkalibacter*, *Methylomicrobium*, and *Roseimarinus* (from the Bacteroidia class). Among the archaea, *Methanoculleus*, *Methanosaeta*, *Methanobacterium*, and Marine Benthic Group D‐and‐DHVEG‐1 stand out with high scores, whereas the most negative scores are attained by *Thiomicrospiria*, *Thalassolituus*, *Alcanivorax*, *Desulfovibrio*, *Desulfofustis*, *Desulfoconvexum*, *Methylomonas* and archaea such as *Halarchaeum* and *Woersearchaeales* (Figure 4). For the model built on Bray‐Curtis distance using genera data from 16S rRNA V3‐V4 sector, one predictor (Cl/HCO_3_) was found to be significant and the constrained axis accounts for merely ~21% of the total variation (Figure 5A). The second in importance, Cl/SO_4_ (non‐significant at *p* = 0.13) and the second constrained axis were included in the ordination solely for comparative purposes with other models (Figure 5A). Remarkably, both log‐ratios exhibit a positive correlation with the RDA1 axis in the ordination, yet they point in opposite directions along the RDA2 axis. The highest scores for the first and only significant axis reveal genera such as *Marinobacter*, *Halomonas*, OPB41, *Sphingomonas*, *Serratia*, and *Halanaerobium*, whereas the lowest scores include *Sulfurimonas*, *Desulfotignum*, Marine Methylotrophic Group 2, *Methylomicrobium*, *Sedimenticola*, *Thiomicrospira*, *Acholeplasma*, and *Alcanivorax*. The most prominent positions in the ordination demonstrate *Marinobacter* and *Halomonas*, whereas on the opposite side, *Sulfurimonas* is notable. The corresponding model based on the V4‐V5 sector data yielded statistical significance with respect to two variables, that is, Cl/SO_4_ and SEC (Figure 5B). Two constrained axes were identified in this case, collectively sharing only 35% of the total inertia. At the first axis, controlled largely by Cl/SO_4_, the highest scores, located in the outlying positions, are associated with the archaea *Methanobacterium* and *Methanocalculus*. Subsequently, much lower values are associated with genera such as *Denitrovibrio*, *Desulfomonas*, *Desulfovermiculus*, *Geopsychrobacter*, *Methanoculleus*, and *Methanohalophilus*. On the negative side of the axis, the most important taxa include *Desulfotignum*, *Candidatus Desulforudis*, *Desulfocarbo*, *Sedimenticola* and a sequence representing family Desulfocapsaceae. Regarding the second axis of the db‐RDA solution, approximated by salinity, *Methanobacterium* received the highest scores, whereas the lowest scores, following the increasing values of SEC, are associated with *Methanocalculus* and *Guyparkeria*.

## DISCUSSION

### Interpretation of gradients and microbial responses in the multivariate ordinations

From the sample size of the analysed data, the db‐RDA solutions reveal only one significantly constrained axis in most cases (Figure 5). The gradients, along which microbial communities align with various configurations (depending on the distance metric employed), are controlled by log‐ratios of the three prevalent anions (Cl, SO_4_, HCO_3_) and SEC (Figure 5). *Guyparkeria*, *Methanocalculus* and, to lesser extent, the uncultured family Desulfotomaculales are mostly associated with high SEC (Figure 5B), usually correlated with high level of TDS. These halophilic bacteria and archaea occur in highly saline environments and are involved in methanogenesis (*Methanocalculus*; Sorokin et al., 2015), sulphate reduction (Desulfotomaculales; Aullo et al., 2013), or sulphur oxidation (chemolithoautotrophic *Guyparkeria*; Lau Vetter et al., 2022). As chloride is highly dominant in the total ion composition (on a par with Na), its proportions to the other two anions can be approximated to the overall sulphate and carbonate prevalence. However, the Cl/SO_4_ ratio has the most significant impact on microbial assemblages across taxonomical levels, particularly due to the strong response observed in archaeal sequences. Here, *Methanobacterium* and *Methanocalculus* seem to proliferate at the low SO_4_ level, whereas *Desulfotignum*, *Candidatus Desulforudis*, and *Thiomicrospira* prefer high concentrations of SO_4_ relative to Cl, as revealed by the V4‐V5 amplicon sequence data (Figure 5). This is consistent with their methanogenesis or sulphate reduction metabolism. These anaerobes are involved in hydrogenotrophic methanogenesis (*Methanocalculus* and *Methanobacterium*; Katayama et al., 2022), sulphate reduction (*Desulfotignum*, Kuever et al., 2015; *Candidatus Desulforudis*, Karnachuk et al., 2019), and sulphur oxidation (*Thiomicrospira*, Brinkhoff et al., 2015). In sequences amplified with starters targeting the V3‐V4 region, diversity is primarily regulated by carbonates (Cl/HCO_3_), whereas the sulphate (Cl/SO_4_) signal appears to be weaker (Figure 5). In this case, the Cl/HCO_3_ ratio is important for the *Halomonas* growth and less so for *Sulfurimonas* and *Desulfotignum* (Figure 5). This consortium of anaerobic bacteria is halophilic (*Halomonas*; de la Haba et al., 2014), reduces sulphates to sulphides (*Desulfotignum*; Qian et al., 2022), reduces nitrate and oxidises sulphur and hydrogen (*Sulfurimonas*, Han & Perner, 2015). *Marinobacter*, a salt‐tolerant genus (Bowman & McMeekin, 2015), is mostly dependent on the Cl/SO_4_ gradient and to a lesser extent on Cl/HCO_3_ (Figure 5A).

A substantial portion of the overall variance in the data, extending along the directions of the unconstrained MDS axis of the db‐RDA analysis on the WGSUniFrac distance, is influenced by factors beyond hydrochemical or by constituents not analysed herein. The most important unconstrained axis of the ordination, contributing 31.7% to the total squared distance, shows the lowest values at the U‐3 and SW‐2 samples, and the highest at the Łapczyca and Tyrawa samples (though the axis direction is arbitrary) (Figure 3A). Regarding the microbial assemblages, along the MDS1 axis, they are ordered with a decreasing number of taxa at all taxonomic levels (Figure 3B). Therefore, this tendency can simply indicate a richness gradient. The gradient crosses the genetic ties of samples, since the SW‐2 and Tyrawa samples represent the flysch aquifer but have opposite positions on the MDS1 axis, which is most likely due to the different genesis of water (the solvent) itself and the components dissolved in it. The impact of groundwater mixing in near‐surface environment on microbial communities of the Tyrawa samples (especially from the Warzelnia spring) seems probable, as shown by sequences representing a very diverse microbial metabolism (Supporting information Figure S3). On the contrary, the U‐3 brine sample was collected from an aquifer at a depth of >1.5 km, isolated from the influence of water from near‐surface environments. Another potential contributor to diversity, of an artificial character, is contamination, which may have randomly occurred despite efforts to fulfil stringent analytical protocols. However, the contamination bias was rather negligible, since human and animal‐related functional pathways were rare and represented by sequences of very low abundance (Supporting information Figure S3).

The db‐RDA solutions derived from the Bray‐Curtis and WGSUniFrac distances differ with respect to the highest genus scores fitted to the ordinations, even when projected onto the same hydrochemical gradients or ordination axes (Figure 3A). Notably, this discrepancy concerns, for example, *Desulfotignum*, *Marinobacter*, and *Sulfurimonas*, which are distinct in the Bray‐Curtis distance mapping of the V3‐V4 gene region data but not in the WGSUniFrac mapping. This arises from the presence of the taxa in all or several samples distributed evenly along the axis but with varying abundances, which is not captured by the WGSUniFrac distance. However, the latter distance metric preserved the most conservative presence–absence responses to environmental gradients in the microbial community, weighted across the taxonomic structure; these responses are herein represented by taxa attributed to the diverse classes such as Desulfuromonadia, Clostridia, Desulfobulbia, Methanosarcinia, Thermoplasmata, and Halobacteria. These anaerobic archaeal‐bacterial communities are mostly salt‐tolerant methanogens, sulphur, and sulphate reducers to H_2_S and acidophiles. Genera such as *Dethiosulfatibacter* (Takii et al., 2007) and *Fusibacter* (Brioukhanov et al., 2023), known for their presence in marine and saline environments and characterised by thiosulphate‐reducing capabilities, alongside extremophiles like *Deferrisoma* (potential iron and sulphur reducer) and deep‐sea, anoxia‐tolerant, sulphate‐reducing MSBL‐8, as well as possibly methanogenic *Smithella*, alkaliphilic and/or iron‐reducing *Alkalibacter*, Sva1033, and *Lacimicrobium*, were typical of environments with the low SO_4_ to Cl ratio. Additionally, alkaliphilic and/or methanotrophic *Methylomicrobium*, *Natronohydrobacter* and archaeal methanogens from the family Methanomethylophilaceae along with other genera such as *Methanoculleus*, *Methanosaeta*, and *Methanobacterium*, are also associated with such conditions. Conversely, environments rich in sulphate are characterised by sulphur‐oxidising microorganisms like *Thiomicrospira*, methanotrophic *Methylomonas*, alkane‐degrading *Alcanivorax*, and sulphate‐reducing *Desulfofustis* and Desulfocapsaceae (Chen et al., 2020; Deja‐Sikora et al., 2019; Li et al., 2019; Scott et al., 2019).

Several other elements, such as K, Ba, Ca, and PO_4_ (Figure 1B), exhibit strong positive or negative correlations with the major ions in the db‐RDA solution (i.e., Cl, SO_4_, and HCO_3_). These correlations suggest possible genetic or formation‐related links between these elements and the major ions. Additionally, they might contribute to microbial processes in the studied groundwaters, especially ions containing biogenic P and K. Even so, their impact is not strong enough to be detected by the linear regression methods in a small sample scenario. This is also true for trace elements forming the I‐Br and Sr‐Li‐B clusters, which are related to each other but not associated with any of the major ions forming significant db‐RDA predictors.

### Functional signatures in the saline and brine groundwater

The structure of microorganisms in the studied saline and brine groundwaters is controlled by the relative concentrations of Cl, SO_4_, and HCO_3_. These ions can exert diverse effects on microbial metabolism, functioning as both cofactors and inhibitors, depending on the specific metabolic context. Chloride is important for maintaining osmotic balance and for the operation of some transport systems in microbial cells (Oren, 2008). Some halophilic enzymes have chloride‐binding sites, which enhance catalytic efficiency and stability (Markússon et al., 2022) or are activated by Cl (Kumar & Khare, 2015). However, at elevated concentrations, Cl can inhibit microbial activity by interfering with redox enzymes and destabilising pH thus affecting functions (Oren, 2008). Sulphate ions, on the other hand, are crucial for the action of sulphotransferases, which catalyse the transfer of sulphate groups to various substrates, playing an important role in the biosynthesis of organic compounds such as glycosaminoglycans. These enzymes participate in numerous biological processes across all living organisms (Paul et al., 2012). However, in certain microorganisms, sulphates can inhibit metabolic processes. In methanogenesis, for instance, elevated SO_4_ levels compete with H_2_ for electrons, thereby reducing CH_4_ production efficiency (Yin et al., 2020). Salinity impacts both sulphur and methane metabolism primarily by affecting the availability of key substrates (such as sulphate, hydrogen and acetate) and imposing osmotic stress on microorganisms. While high salinity may enhance sulphur metabolism by increasing sulphate availability, it can inhibit methanogenesis by favouring sulphate‐reducing bacteria over methanogens.

Bicarbonate ions, on the other hand, are important in enzymatic reactions involving carbonic anhydrase, enzymes catalysing reversible hydration/dehydration reactions, converting CO_2_ into bicarbonate ions while releasing protons. This action is crucial for maintaining acid–base balance and CO_2_ transport (Zaidi et al., 2022). Additionally, in autotrophic microorganisms, HCO_3_ serves as a carbon source in the Calvin cycle during photosynthesis and chemosynthesis.

Metabolic and ecologically relevant functions of microorganisms in the studied samples were determined using 16S RNA amplicons to generate functional profiles through FAPROTAX as proposed by Louca et al. (2016). Each function is characterised by its primary energy source (carbon, nitrogen, sulphur, arsenic, and others). The carbon cycle functional groups vary depending on the sample location, leading to the identification of several chemoheterotrophic groups. The first group is dominated by fermentation and hydrocarbon degradation activities. The second group consists of potential heterotrophs that utilise carbon compounds other than lignin, chitin, xylan, cellulose, methanol, methane, and aromatic hydrocarbons, and are classified as aerobic chemoheterotrophs. Additionally, in the SW‐2 sample, the hydrocarbon degradation group predominantly comprises methane‐oxidising bacteria, allowing the distinction of a methanotrophy functional group (Figure 3C). Analysis of the V4‐V5 region sequences indicates that methanogenesis is a prevalent function among microorganisms in many samples (Łapczyca, Zabłocie and to some extent Tyrawa Solna). Specifically, samples from Łapczyca are characterised by methanogenesis utilising formate and hydrogenotrophic methanogenesis (*Methanocalculus* and *Methanobacterium*), whereas the Zabłocie sample exhibits only hydrogenotrophic methanogenesis. To some extent (mainly in sample B‐19 from Busko), methanogenesis via reduction of methyl components with hydrogen (*Methanohalophilus*) is also predicted (Figure 3C). In sulphate‐rich environments (such as Busko groundwaters), hydrotrophic methanogens compete with sulphate‐reducing bacteria for H_2_. However, methylated substrates that are not utilised by sulphate‐reducing bacteria are often abundant in brines and promote methylotrophic methanogenesis (Saxton et al., 2021).

These different pathways of CH_4_ generation show that methane is chiefly produced here by methanogens which use hydrogen (hydrogenotrophs) and formate as electron donors to reduce CO_2_ to CH_4_ in six steps via the reductive acetyl‐CoA or Wood‐Ljungdahl pathway (Liu & Whitman, 2008; Thauer et al., 2008), although the proportions vary between the analysed samples.

The most abundant OTUs related to the nitrogen cycle (nitrogen fixation and respiration, nitrate and nitrite ammonification, nitrate reduction) were detected in the Warzelnia and Zabłocie samples (Figure 3C). Despite the different requirements of the various microorganisms involved in these processes (e.g., oxygen requirements), many nitrogen cycle reactions co‐occur in the natural environment (Kuypers et al., 2018; Mosley et al., 2022). In the Warzelnia and Zabłocie samples, bacteria from the genera *Marinobacter* and *Denitrovibrio* are particularly involved in the nitrogen cycle (Kiss et al., 2010; Li et al., 2013). Moreover, methanogens such as *Methanobacterium*, which can also participate in nitrogen metabolism, have been found in these waters (Figures 2B and 3C; Bae et al., 2018).

Taxonomic and functional analysis showed that in the samples collected from Busko, a significant proportion of the microorganism community is the one involved in the sulphur cycle, encompassing both reduction and oxidation processes of sulphur compounds (Figure 3C). In contrast, in samples from Łapczyca and Tyrawa Solna, the proportion of sulphur bacteria is less substantial and they are mainly associated with the reduction of sulphur compounds. This is reflected in the db‐RDA ordination (Figure 3A). Specifically, in B‐19, which has the highest sulphate concentrations (2677 mg/L), the functional groups associated with sulphate oxidation (dark sulphur/sulphide oxidation) and reduction of sulphur compounds (respiration of sulphur compounds) are the most representative (Figure 3C).

Metabarcoding taxonomic assignment indicates that the brine microbiome is dominated by genera capable of reducing sulphate to H_2_S, such as *Desulfotignum*, *Desulfocarbo*, and uncultured Desulfotomaculales, with a total relative abundance exceeding 57% (Figure 2B). In the Busko samples, 4 and 23% of the readings are assigned to the genus *Desulfotignum* (Figure 2B). These anaerobic chemoorganotrophs/chemoautotrophs use formate, acetate, butyrate, higher fatty acids, other organic acids, alcohols, or similar aromatic compounds as carbon sources and electron donors. In the absence of organic carbon, they can also perform chemolithoautotrophy, using H_2_ as an electron donor, while utilising sulphates, sulphites and thiosulphates as terminal electron acceptors (Kuever et al., 2001). Conversely, in B‐19, approximately 22% of the reads were assigned to the genus *Thiomicrorhabdus* (previously identified as *Thiomicrospira*) (Figure 2B), anaerobic chemolithoautotrophs that use reduced, inorganic sulphur compounds such as S^0^, sulphide, tetrathionate, thiosulphate (but not sulphite or thiocyanate), and CO_2_ as a carbon source. The final product of oxidation is sulphate. Therefore, the analysed samples contain both the substrates and products of sulphur compound reduction and oxidation processes (Brinkhoff et al., 2015). However, the dynamics and trajectory of thiosulphate cycling may vary, as suggested by the absence of *Dethiosulfatibacter* and *Fusibacter* in the most sulphate‐rich samples where *Thiomicrospira* is present, and vice versa, a finding also captured by the binary db‐RDA ordination (Figures 3A and 4).

The abundance of sulphur‐cycling chemolithoautotrophic microbial communities in B‐19, along with their metabolic plasticity, indicates that sulphide production and preservation in these types of environments is likely quite common. This relationship has also been demonstrated in other halophilic environments (Fairén et al., 2023; Magnuson et al., 2023; Pontefract et al., 2017; Stam et al., 2010; Torfstein et al., 2005), which are considered astrobiologically as temporal analogues for Mars.

Analyses of other sulphate‐rich waters or sediments (Bell et al., 2020; He et al., 2020; Holmkvist et al., 2011; Mills et al., 2016; Payler et al., 2019; Sabuda et al., 2020) show that extensive sulphur cycling occurs in these environments. Moreover, it has been shown that even despite limited hydrogeochemical evidence for sulphate reduction, microorganisms use sulphur compounds for energy.

### Distribution of methane related microbes

Life of microorganisms, including those associated with methane in the analysed samples, is limited by the presence of salt (as mostly expressed by log‐ratios of Cl, SO_4_, HCO_3_, and SEC), but also determined by low oxygen content. The functioning of microorganisms in the isolated surface of low‐oxygen groundwater depends on the use of available electron donors and acceptors (Dowd et al., 2022). The conditions that determine microbial life vary depending on the origin of the groundwaters. Both taxonomic and functional analyses indicate that aerobic oxidation of methane occurs mainly in SW‐2 brine, which has the highest Eh (+193 mV) and relatively low salinity (SEC = 65.2 mS/cm) compared to the other analysed waters. This is because this brine comes from a shallow borehole from a depth of 57 m. It is sedimentary water diluted with dehydration waters (Rajchel et al., 2004). Moreover, methane has been found in Sól brines (Rajchel et al., 2004), which is the substrate for the identified methane‐oxidising microorganisms. Nevertheless, such a value of the reduction potential (+193 mV) is indicative of the low oxygen content of this environment and the identified methanotrophs and methylotrophs are adapted to such conditions. Marine Methylotrophic Group 2 (also referred as deep‐sea clade 2; Parks et al., 2018) and *Methylomicrobium* play major roles as anaerobic methane‐oxidising bacteria in this environment. Marine Methylotrophic Group 2 lives in symbiosis with cold‐seep sponges (Wang, Gong, et al., 2023; Wang, Liu, et al., 2023) or occurs as free‐living bacteria in methane‐rich and low oxygen environments, such as deep‐sea sediments, water column of vents, hydrothermal fields and cold seeps (Ruff et al., 2013). *Methylomicrobium* is an alkaliphilic and halotolerant methanotroph (Bordel et al., 2020), which can grow at low‐oxygen tension and display fermentation and denitrification capabilities (Kalyuzhnaya, 2015; Stone et al., 2020).

In contrast, methane‐forming processes prevailed in samples from Łapczyca and Zabłocie. These brines are relic Miocene marine waters. Unlike the SW‐2 brine, they are not diluted by other water components, and their composition is similar to ocean water (Zuber & Grabczak, 1985). Łapczyca and Zabłocie brines are characterised by similar chemistry (Figure 1C), with Eh ranging from +16 to +129 mV and salinity up to 188 mS/cm (Supplementary material Table S1). At the same time, it is understood that different environments exhibit varying critical redox potential values for methanogenesis (Szafranek‐Nakonieczna & Stępniewska, 2015). Methanogenesis in a high TDS groundwater is a complex process that can be significantly different from methanogenesis in other environments. The analysed waters contain large amounts of mineral compounds, such as chlorides, sulphates, hydrogen carbonates, and so forth (Supplementary material Table S1). These compounds can affect the availability of substrates and redox processes taking place in the environment. In the brines from Zabłocie and Łapczyca, methanogenesis is carried out by halotolerant and halophilic methanogens from the genera *Methanocalculus*, *Methanobacterium*, and *Methanohalophilus*.

**FIGURE 6:**
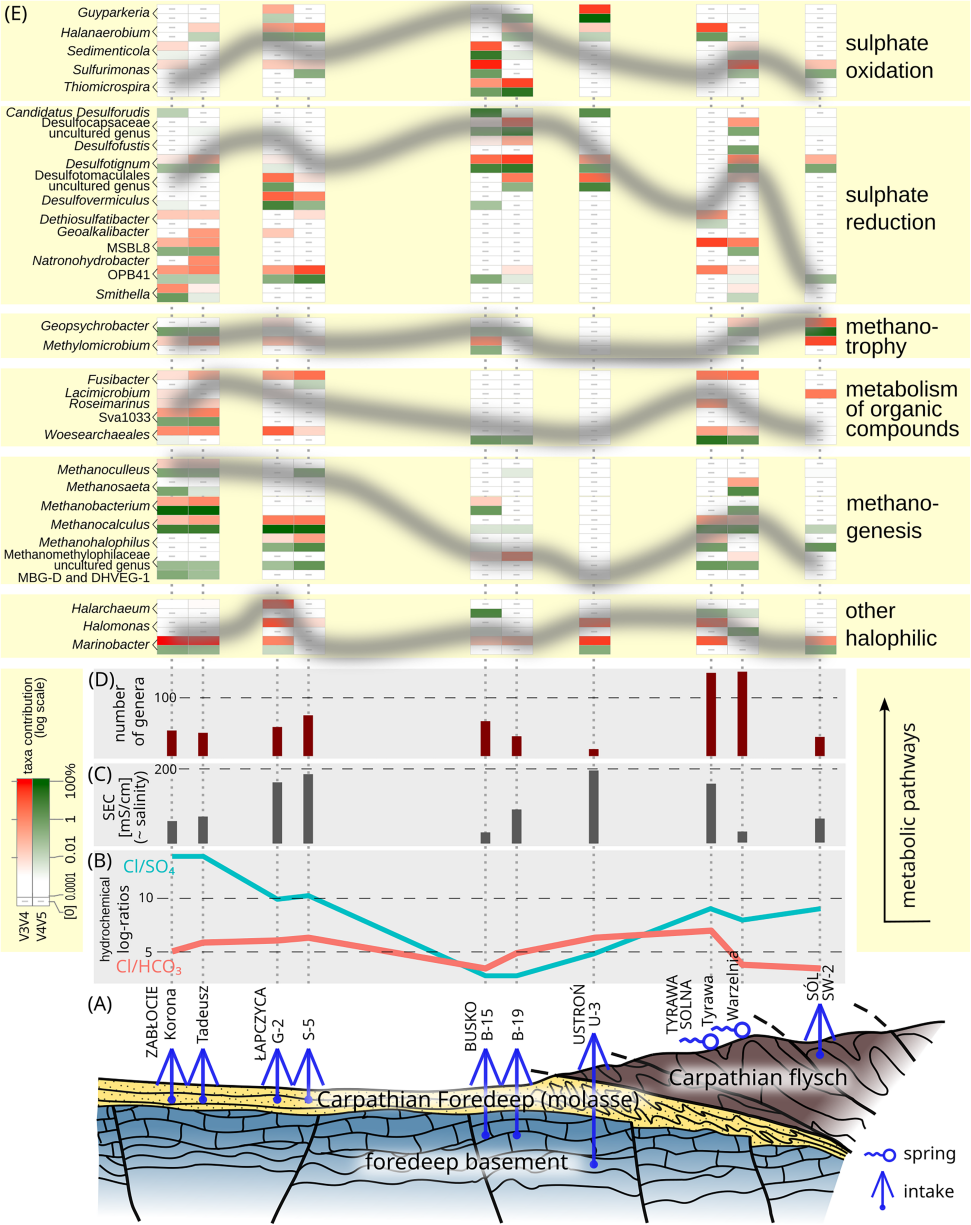
Overview of microbial processes and hydrochemical factors in brine and saline groundwaters of southern Poland. (A) Schematic geological cross section illustrating the position of groundwater within the main aquifer types studied. (B, C) Key hydrochemical predictors from the RDA models: Hydrochemical log‐ratios (B), and specific electrical conductivity (SEC) (C). (D) Genera richness and (E) selected microbial taxa with typically high scores on the RDA models, categorised by their likely dominant metabolic functions. Faded grey lines indicate possible, approximate, and relative contributions of these categories to microbial community structure across the samples. Red and green shades represent the percentage of microbial sequences amplified with starters targeting V3‐V4 and V4‐V5 gene regions, respectively.

Methane oxidation by methanotrophs is a more salinity‐sensitive process than methane production by archaea. For most conventional methanotrophs, salinity levels of ~5–10 g/L NaCl can already inhibit their growth and metabolism. However, some halotolerant methanotrophs like species of the genus *Methylomicrobium* can tolerate salinities of up to 90 g/L NaCl (Bordel et al., 2020). This genus, co‐occurring with the anaerobic methane oxidiser *Geopsychrobacter*, tends to proliferate in samples with moderate salinity (18–40 g/L NaCl) from Sól, Busko, and Zabłocie, but is also observed in Łapczyca at 134 g/L NaCl (Figures 2 and 6). The latter anomaly can be explained by an influx of methanotrophs (possibly inactive cells) from environments with lower salinity through water mixing. For most methanogens, the critical salinity is ~15–20 g/L NaCl. Above this level, their ability to produce methane is significantly inhibited. However, in the case of methanogens, there are more taxa that are halotolerant and halophilic. Halotolerant archaea, such as *Methanobacterium* and *Methanocalculus* can survive in environments with salinities up to 85 g/L NaCl (Zhang et al., 2017) and 125 g/L NaCl (Garrity et al., 2001). *Methanohalophilus*, another extremophilic halophilic methanogen, can survive in environments with salinities as high as 180 g/L NaCl (Christman et al., 2020) and even up to 240 g/L NaCl (L'Haridon et al., 2020). Salinity tolerance of these, along with other halophilic archaea (such as *Halarchaeum*) and bacteria (e.g., *Halanaerobium*, *Guyparkeria*), is reflected in their abundance across various TDS (and SEC) levels (Figure 6). The halophilic tendencies of archaea can also be linked to their positioning along the RDA 2 axis in the RDA ordination (Figure 5B).

## SUMMARY

The key findings of this study are summarised as follows:

- The correlation between hydrochemical ions in the water samples unveils significant associations, indicating factors such as the origin of relic groundwaters, their interaction with aquifer rocks (containing, e.g., sulphate, chloride and carbonate‐phosphate solid phases), and ion pair precipitation. Three distinct groups are identified in the hydrochemical composition of the samples, each linked to a specific geological structure in southern Poland, that is, Carpathian flysch, Carpathian Foredeep, and its basement.
- The Cl/SO_4_ and Cl/HCO_3_ log‐ratios, along with SEC evaluating overall salinity, constitute significant environmental gradients associated with microbial communities in db‐RDA ordinations, derived from cross‐taxonomic, composite bacterial‐archaeal binary WGSUniFrac distance and the abundance‐oriented Bray‐Curtis distances. However, these predictors account for only a small proportion of the total inertia, which may be attributed to relatively weak environmental signals and a limited sample size.
- A striking association is identified between microbial sulphur cycle activity and the SO_4_ to Cl ratio, with the highest levels observed in samples from Busko and the lowest recorded in Zabłocie (Figure 6). Sulphate‐reducing microorganisms tend to mirror the presence of sulphate‐oxidisers, implying the availability of substrates and products for both processes.
- In contrast, methanogens and microorganisms metabolising organic compounds dominate in samples with low to moderate sulphur metabolism (such as Łapczyca and Tyrawa) (Figure 6), suggesting competition for electrons between sulphate and hydrogen in the groundwater system.
- Groundwater characterised by an elevated HCO_3_ to Cl ratio (in Busko particularly B‐15, Warzelnia in Tyrawa Solna, and Sól) is likely involved in hydrocarbon degradation pathways, coupled with methanotrophy and sulphur cycling.
- Groundwater from the Carpathian flysch (particularly from Warzelnia) exhibits more complex dynamics, likely influenced by nitrogen cycling, whereas the microbial communities in the deepest intake (U‐3) are challenging to interpret due to the low DNA content of the water sample.
- The proportion of halophilic bacteria and archaea generally increases with overall salinity, which is proxied by SEC. However, salinity appears to regulate the distribution of specific methanotrophs and methanogens.

## CONCLUSION

The geochemical composition of the groundwater samples reveals three distinct clusters corresponding to specific geological environments in southern Poland, which define the groundwater's origin. The log‐ratios involving Cl, SO_4_, and HCO_3_ ions, and SEC, are identified by db‐RDA ordinations based on binary and abundance‐oriented distance metrics as being strongly associated with microbial communities. Sequencing of the V3‐V4 and V4‐V5 gene regions, along with functional analysis, provides a comprehensive insight into the taxonomy and metabolism of these microbial communities, encompassing bacterial methanotrophs and archaeal methanogens engaged in methane transformation within saline and brine groundwaters. The primary microbiological processes observed in these samples include hydrogenotrophic methanogenesis, methanotrophy, sulphate reduction, and sulphur oxidation, all performed by salt‐tolerant and halophilic microorganisms. These findings suggest that analysing the microbial structure of saline and brine groundwaters with varying hydrochemical properties can provide valuable information on microbial life in extreme habitats, both on Earth and on other planets (e.g., Mars, Saturn's moons), as well as early sulphate ecosystems on Earth.

## AUTHOR CONTRIBUTIONS

**Dariusz Dobrzyński:** Resources; writing – review and editing. **Mirosław Słowakiewicz:** Conceptualization; writing – original draft; funding acquisition; resources; investigation; project administration. **Tomasz Segit:** Writing – original draft; software; formal analysis. **Katarzyna Wątor:** Resources; writing – review and editing. **Weronika Goraj:** Investigation; writing – original draft; resources; formal analysis.

## CONFLICT OF INTEREST STATEMENT

The authors declare no conflict of interest.

## Supporting information


**Data S1** Supporting Information.

## Data Availability

The data that support the findings of this study are openly available at https://www.ncbi.nlm.nih.gov/bioproject/PRJNA1045246.
