# Supplementary material for: Hydrochemical gradients driving extremophile distribution in saline and brine groundwater of southern Poland
Source: Environ Microbiol Rep. 2024 Oct 23;16(5):e70030. doi: 10.1111/1758-2229.70030 (PMC11497496; doi:10.1111/1758-2229.70030)
Supplement: Supplementary file 1 — Data S1 Supporting Information. [file EMI4-16-e70030-s001.docx]

Hydrochemical gradients driving extremophile distribution in the saline and brine groundwater of southern Poland

Mirosław Słowakiewicz^*1^, Weronika Goraj^2^, Tomasz Segit^1^, Katarzyna Wątor^3^, Dariusz Dobrzyński^1^

^1^Faculty of Geology, University of Warsaw, Warsaw, Poland

^2^Faculty of Medicine, The John Paul II Catholic University of Lublin, Lublin, Poland

^3^Faculty of Geology, Geophysics and Environmental Protection, AGH University of Krakow, Kraków, Poland

^*^corresponding author: [m.slowakiewicz@uw.edu.pl](mailto:m.slowakiewicz@uw.edu.pl), [m.slowakiewicz@gmail.com](mailto:m.slowakiewicz@gmail.com)

**OUTLINE OF GEOLOGICAL AND HYDROGEOLOGICAL SETTINGS**

The studied groundwater originates from sites situated in the Outer Carpathians and the Carpathian Foredeep basin in southern Poland (Fig. S1). The Outer Carpathians, representing the youngest and outermost zone of the Alpine fold-and-thrust belt of the Carpathians, primarily consist of turbidite flysch sequences (alternating shale, claystone, mudstone, sandstone and conglomerate), as well as less common siliceous rocks, marl and limestone. In the studied region, these deposits typically represent Upper Cretaceous-Palaeogene sequences. To the north and north-east of the Carpathians, the Carpathian Foredeep basin was formed during the final stage of the Alpine orogeny, as a result of the thrust loading on the foreland plate. In Poland, this unit attains a thickness of up to 3000 m and the basin is mostly filled with the Miocene molasse succession of detrital and chemical sediments. These foredeep deposits overlie older basement rocks of diverse age and origin, ranging from Precambrian crystalline rocks to Cretaceous strata of epicontinental facies.

Both the Outer Carpathians and Carpathian Foredeep basin exhibit groundwater with a broad range of total dissolved solids (TDS), spanning from fresh groundwater (with a TDS of less than 1 g/L) to brines. In the Outer Carpathians, the proportions between sandstone and conglomerate rocks versus claystone and mudstone in the flysch sequences strongly influence the spatial variation of hydrogeological conditions. The contrasting lithology, coupled with intense tectonic disturbances, yield diverse conditions for groundwater occurrence and circulation. Consequently, fresh groundwater commonly co-occurs with various mineral waters (groundwater with TDS >1 g/L). Mineral waters, including brines, tend to ascend through the network of tectonic fissures and mix with fresh groundwater. The active turnover zone of usually fresh groundwater in the Outer Carpathians is estimated to extend to a depth of ~100 m.

The spatial relationship between fresh and mineral waters in the Carpathian Foredeep basin differs from the Outer Carpathians due to the distinct tectono-sedimentary architecture of the Neogene basin fill. Fresh groundwater occurs in Quaternary formations, primarily in river valleys, and in the uppermost part of Miocene formations. The thickness of the freshwater zone in Quaternary aquifers and in the upper part of Miocene formations typically does not exceed 20 m and 30 m, respectively. Saline (TDS between 1 and 35 g/L) and brine (TDS > 35 g/L) waters occur in Miocene formations as well as in older formations within the basin. In the zone along the edge of the Carpathian overthrust, the Miocene formations underwent intense tectonic deformation (Malinowski, 1991; Paczyński and Sadurski, 2007a, 2007b).

For the purpose of this study, relic groundwaters with exceptionally high TDS, indicative of different geological and geochemical conditions in the Outer Carpathians and Carpathian Foredeep basin, were carefully selected. High TDS groundwaters representative of the Outer Carpathians were obtained from Tyrawa Solna and Sól. In Tyrawa Solna, saline and brine waters were also collected from two natural outflows, which unlike carbonated waters, are uncommon in the Carpathians. These waters flow out of the Menilite Beds (chert) and Kliwa sandstone of the Skole unit, near the overthrust of the Sub-Silesian unit (Fig. S1; Szymakowska, 1960; Rajchel, 2016). Their origin has not been investigated yet. The brine in Sól comes from a shallow SW-2 borehole (57 m deep) and is associated with the Ropianka Beds and variegated shales of the Magura unit (Rajchel *et al.*, 2004; Felter *et al.*, 2021). This brine is believed to be original connate water, with its composition subsequently modified through processes of ultrafiltration and dilution with dehydration waters (Rajchel *et al.*, 2004).

The other waters under study originate from Miocene formations in the Carpathian Foredeep basin and its basement rocks. Zabłocie and Łapczyca brines represent the former unit. In Zabłocie, brines associated with silty marly clays with marly sands of the Skawina Formation were sampled from two intakes (Korona, Tadeusz). Brine inflows to the Tadeusz borehole occur in several intervals between 312 and 745 m (Tatarski and Iwanowski, 1976), whereas inflows to the Korona borehole likely took place at depths between 300 and 600 m (R. Ślaski, personal communication). In Łapczyca, brines occurring in the Miocene aquifer at the depth between 400 and 1125 m (Felter *et al.*, 2021), were sampled from two boreholes (S-5 and G-2). According to their O and H stable isotope compositions, both Zabłocie and Łapczyca groundwaters are considered relic Miocene marine waters (Zuber and Grabczak, 1985). The isotopic composition of the brines from Łapczyca slightly deviates from ocean water, presumably due to the ultrafiltration. The Busko brines from intakes B-15 and B-19 occur in the Kimmeridgian (Jurassic) limestone and marl, at depth intervals of 441–500 and 356–600 m, respectively (Krawczyk *et al.*, 1999). They are waters of meteoric origin recharged during the pre-Pleistocene period, probably in the upper Miocene (Zuber *et al.*, 1997). The brine collected from the U-3 intake in Ustroń occurs in Devonian limestone and dolomite at a depth range of 1550–1728 m. This brine, like others present in the Carboniferous strata of the Upper Silesia Coal Basin, primarily represents Early Permian meteoric waters, infiltrated during a hot and dry climate with periodically high rainfall. The salinity source of these waters is attributed to leaching of weathered rocks and enrichment by long-term ultrafiltration (Różkowski and Przewłocki, 1987; Różkowski, 1994; Pluta and Zuber, 1995).

**MATERIALS AND METHODS**

**Chemical analysis of water**

Inductively coupled plasma-optical emission spectrometry (ICP-OES, Optima 7300DV, Perkin Elmer, USA) and inductively coupled-plasma mass spectrometry (ICP-MS, iCAP RQ (C2), Thermo Scientific, USA) were used for analysis of major and trace elements. The procedure described in the appropriate ISO standards (ISO, 2007, 2016) was used. Br and I were determined according to an internal laboratory procedure with the use of ICP-MS. All samples were diluted to avoid oversaturation of the analytical signal and to minimise the occurrence of interferences. The external 5-point calibration curves were established on the basis of properly prepared calibration solutions (multi- and single- element standard solutions from Sigma Aldrich, USA, were used). Chlorides (Cl) and hydrogen carbonates (HCO_3_) were determined with classical titration methods (ISO, 1989, 1994). The implemented QA/QC programme includes duplicate analyses of the same sample and analyses of deionised water samples, standard solutions and certified reference materials with an environmental matrix (HAMIL-20.2 and TMDA-64.3, Canada).

**Hydrochemical and sequence data analysis**

Data manipulation was carried out in R (v. 4.3.2) programming environment (R Core Team, 2023) and most of the graphics were produced using commands in R package ggplot2 (Wickham, 2016), metacoder (Foster *et al.*, 2017) and corrplot (Wei and Simko, 2021). Hydrochemical data analyses were conducted in accordance with the compositional nature of the ion concentrations (Buccianti and Pawlowsky-Glahn, 2005) and the constraint of a small sample size. Imputation of two values below the detection limit employed the imputeBDLs function of the robCompositions R package (Templ *et al.*, 2011) using partial least squares method. The pairwise relationships between variables were computed through Spearman’s correlation coefficients, based on symmetric pivot coordinates (Kynčlová *et al.*, 2017). To address potential data quality issues and inflated variation of components with very low contributions (e.g., values imputed and being at the detection limit), modest coefficients weighting was applied (tuned by a powering parameter k = 0.5), which was based on compositional variation (Hron *et al.*, 2021). The significance of correlation coefficients was tested by a permutation test at P-value threshold of p = 0.05. The correlation distance was mapped onto a 2-dimensional representation using principal coordinate analysis (PCoA; function wcmdscale of the vegan R package, ver. 2.6-4, Oksanen *et al.*, 2022), with compositional variation values (Aitchison, 1982), additionally supplied to the ordination plot. To further examine variable associations, agglomerative hierarchical clustering was performed using the average linkage method (hclust() function). Centred-log-ratio coordinates, weighted by the percentage contribution of components in CLR function of the easyCODA R package (Greenacre, 2018), were utilised for agglomerative hierarchical clustering with the Ward’s linkage method.

Following the processing of raw sequence reads, 16S rRNA data are typically represented as high-dimensional, zero-inflated count tables with hierarchical, phylogenetic, and/or taxonomic structures. To address unclassified sequences, filter out mitochondria and chloroplast reads, and plot taxonomic trees, the R packages, phyloseq (McMurdie and Holmes, 2013) and metacoder (Foster *et al.*, 2017), were utilised. The challenge of data sparsity arose during the acquisition of log-ratio coordinates for compositional analysis, primarily due to bias introduced during imputation for zero-inflated data. Consequently, two alternative scenarios in the processing workflow were employed to gain insights into the data structure under small sample constraints. In the first approach, commonly utilised in microbiological studies (Paliy and Shankar, 2016), abundance data at genus taxonomic level underwent Hellinger transformation to mitigate the relative influence of dominant genera, particularly in light of the stringent filtering applied to raw sequences. Then, Bray-Curtis dissimilarity was calculated. In the second, complimentary and generalised approach, instead of focusing on abundance, inter-sample diversity in the occurrence data was leveraged using the WGSUniFrac metric (Wei and Koslicki, 2022). This method, an extension of the widely used UniFrac distance (Lozupone and Knight, 2005), is noteworthy for its ability to account for the taxonomic tree structure. To take advantage of the targeting various 16S sectors and utilise diverse amplification efficiencies for specific domains, bacterial sequences from the V3-V4 sectors and archaeal sequences from the V4-V5 sectors were combined in advance. WGSUniFrax distance matrix was adjusted with parameter k = -1 to assign slightly more weight towards higher taxonomic levels (Wei and Koslicki, 2022).

To assess the strength of association between microbial communities and hydrochemical variables, the distance-based Redundancy Analysis (db-RDA) was employed. Redundancy analysis, a type of direct gradient analysis, describes each species' abundance as a linear function of measured environmental variables, such as hydrochemical factors (Ter Braak and Prentice, 1988). In this context, gradients may be referred to as variables or their combinations (i.e. ordination axes) that control the distribution and abundance of taxa. Explanatory variables were represented by weighted pairwise log-ratios of ion concentrations (Greenacre and Lewi, 2009), calculated in a non-amalgamated form for ease of interpretation. The selection of features explaining maximum variance (Greenacre, 2019) was performed using the STEP() function of the easyCODa R package (Greenacre, 2018). Only five log-ratios, capturing majority of the total inertia, along with three geochemical parameters (specific electrical conductivity, temperature and pH), were included in the initial model. This limitation was attributed to the small observations to variables ratio. The final model was constructed using a forward variable selection algorithm (ordiR2step function of the vegan package, Oksanen *et al.*, 2022). Formal significance testing of ordinations, constrained axes, and explanatory variables was performed with permutation tests of anova.cca function of the vegan R package. To enhance interpretability of the db-RDA solution for WGSUniFrac distance, abundance data, representing taxonomic ranks from genus to class, were mapped onto the db-RDA solution, with reservations about the projection accuracy due to the use of a non-Euclidean distance (see details of sppscores function, vegan package, Oksanen *et al.*, 2022). Here, the 16S gene regions for metabarcoding were utilised, which in contrast to shotgun metagenomics, cannot directly supply information on the metabolic traits of the microbiome. To address such limitations, various tools have been developed to map barcoding-derived taxonomic identification to functional groups. These tools involve browsing databases containing functional annotations of cultured microorganisms, drawing on existing literature. The FAPROTAX database was utilised, which functionally classified bacteria and archaea associated with the marine environment (Louca *et al.*, 2016). Since analysis of high TDS groundwater with a probable pristine marine genetic component was the main focus, the FAPROTAX database appeared to be the most relevant reference herein. However, it is important to stress certain limitations and assumptions associated with the tool, such as: i/ assuming equal functional abilities for cultured and uncultured members of the same taxon, ii/ the possibility of human errors present in the literature data being transferred to the database, iii/ current inability to associate many known taxa with any function, and iv/ the absence of additional functions, if they were assigned to a taxon following the database creation (Louca *et al.*, 2016). Raw (unfiltered) OTUs for predicting microbial functional groups were supplied on the basis of annotations stored in the FAPROTAX database v. 1.2.4. by means of the microeco R package (Liu *et al.*, 2021).


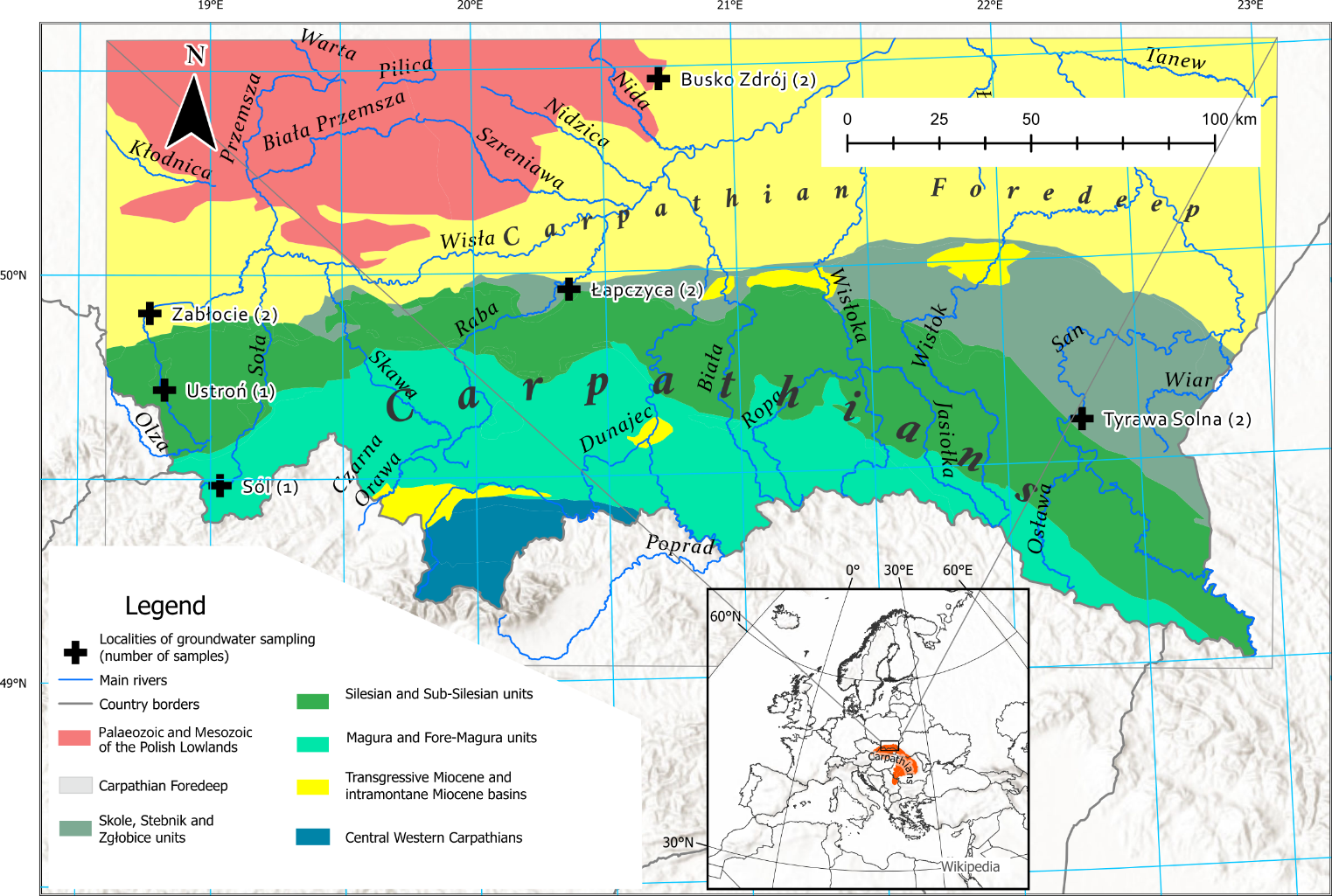


Fig. S1. Location of groundwater sampling sites in relation to the geological structures (after Oszczypko and Zuber, 2002).

Fig. S2. The percentage of sequences classified as bacterial, archaeal, and unclassified in datasets generated using primers targeting the V3-V4 and V4-V5 regions of the16S rRNA gene.


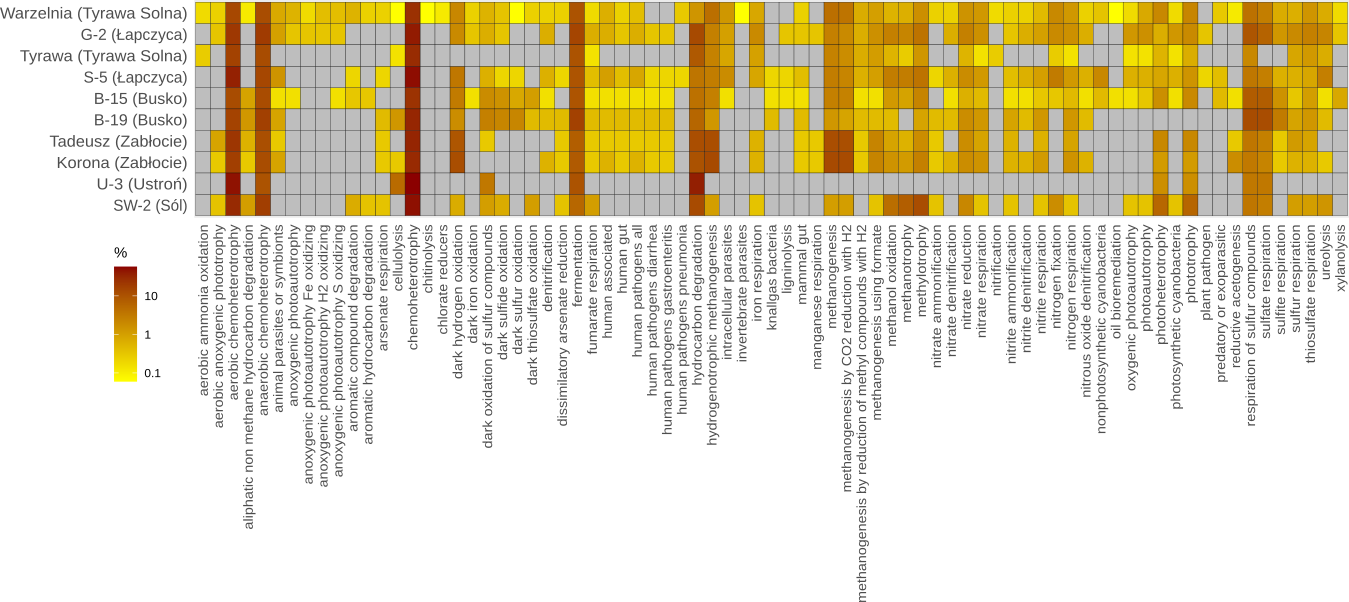


Fig. S3. Percentage of OTUs (unweighted by abundance) with specific trait in communities, inferred from FAPROTAX database (Louca *et al.*, 2016) using unfiltered, combined sequencing data of from the V3-V4 and V4-V5 gene regions; rows (samples) ordered along MDS1 axis of the dbRDA solution for WGSUniFrac distance (see Fig. 3A).

Table S1. Physicochemical parameters of the studied saline and brine groundwaters. Concentrations in mg/L unless otherwise indicated. TDS – total dissolved solids; SEC – specific electrical conductivity; E_H_ – redox potential corrected with standard hydrogen electrode (SHE); PCC – prevalent chemical character (hydrochemical type based on ions with > 20% gram equivalent); na – not analysed.

|  | Korona (Zabłocie) | Tadeusz (Zabłocie) | SW-2  (Sól) | G-2  (Łapczyca) | S-5  (Łapczyca) | B-15  (Busko) | B-19  (Busko) | Warzelnia (Tyrawa Solna) | Tyrawa  (Tyrawa Solna) | U-3  (Ustroń) |
| --- | --- | --- | --- | --- | --- | --- | --- | --- | --- | --- |
| Date of sampling | 16.02.2021 | 16.02.2021 | 25.05.2021 | 26.05.2021 | 26.05.2021 | 17.02.2021 | 17.02.2021 | 28.05.2021 | 28.05.2021 | 01.04.2022 |
| Coordinates | 49^o^54’27.93”N; 18^o^46’14.15”E | 49^o^54’26.16”N; 18^o^46’11.91”E | 49^o^29’09.36”N; 19^o^02’10.89”E | 49^o^56’58.94”N; 20^o^21’01.43”E | 49^o^57’30.10”N; 20^o^20’52.09”E | 50^o^27’30.58”N; 20^o^43’01.91”E | 50^o^27’14.76”N; 20^o^43’40.59”E | 49^o^36’13.86”N; 22^o^16’47.45”E | 49^o^36’12.36”N; 22^o^17’04.45”E | 49^o^43’11.66”N; 18^o^49’36.34”E |
| Temperature [°C] | 11.7 | 11.9 | 11.1 | 19.7 | 12.7 | 13.0 | 11.5 | 8.6 | 13.9 | 15.9 |
| TDS [g/L] | 41.530 | 50.479 | 43.233 | 131.625 | 151.159 | 20.466 | 69.006 | 22.074 | 139.986 | 95.779 |
| SEC [mS/cm] | 61.4 | 73.3 | 65.2 | 166.0 | 188.2 | 30.3 | 92.5 | 30.0 | 161.9 | 198.2 |
| pH | 7.25 | 7.79 | 7.33 | 6.98 | 7.31 | 8.22 | 7.10 | 6.94 | 7.09 | 6.89 |
| E_H_ (SHE-corrected) [mV] | 129 | 16 | 193 | 128 | 98 | -37 | 136 | 124 | 103 | 93 |
| Na | 13446 | 15838 | 15607 | 45988 | 52883 | 6991 | 21354 | 7306 | 46192 | 23629 |
| K | 96.63 | 113.4 | 62.47 | 223.3 | 240.9 | 130.3 | 403.6 | 144.1 | 281.9 | 492 |
| Li | 4.010 | 4.585 | 23.22 | 6.953 | 5.795 | 1.866 | 6.515 | 5.169 | 45.63 | 9.732 |
| Be | <0.0005 | <0.0005 | 0.0028 | 0.0008 | 0.0069 | <0.0005 | <0.0005 | <0.0005 | <0.0005 | <0.0005 |
| Ca | 1146 | 1707 | 258.9 | 1919 | 2440 | 177.8 | 2407 | 679.2 | 6536 | 7784 |
| Mg | 584.7 | 766.8 | 203.5 | 1303 | 1444 | 301.2 | 1294 | 83.4 | 818.8 | 2386 |
| Ba | 29.47 | 46.05 | 123.2 | 81.07 | 88.39 | 0.0574 | 0.0513 | 19.48 | 96.13 | 1.95 |
| Sr | 29.89 | 46.54 | 111.9 | 100 | 149.8 | 48.95 | 43.74 | 72.04 | 604.4 | 326.3 |
| Fe | 9.08 | 25.16 | 0.51 | 18.92 | 21.38 | 13.82 | 33.01 | 10.86 | 15.54 | 13.72 |
| Mn | 1.103 | 1.158 | 0.03 | 1.422 | 2.272 | 0.398 | 0.41 | 0.533 | 11.5 | 0.648 |
| Ag | 0.005 | 0.021 | 0.007 | 0.007 | 0.014 | <0.001 | <0.001 | 0.002 | 0.002 | 0.058 |
| Zn | 0.08 | 0.01 | 0.03 | <0.01 | 0.42 | 0.015 | 0.02 | 0.13 | 0.16 | <0.01 |
| Cu | 0.001 | <0.001 | 0.104 | 0.037 | 0.1 | 0.005 | <0.001 | 0.019 | 0.027 | <0.05 |
| Ni | <0.003 | 0.002 | 0.012 | 0.006 | 0.026 | <0.001 | 0.004 | 0.03 | 0.024 | <0.05 |
| Co | 0.0005 | <0.0002 | 0.0042 | 0.0016 | 0.0099 | 0.0003 | 0.0009 | 0.0018 | 0.0026 | 0.0015 |
| Pb | <0.0001 | <0.0001 | 0.0026 | 0.0017 | 0.0344 | <0.0001 | <0.0001 | 0.0065 | 0.0047 | <0.005 |
| Hg | <0.0001 | <0.0001 | 0.0438 | 0.0582 | 0.0675 | <0.0001 | <0.0001 | 0.0007 | 0.0003 | <0.05 |
| Cd | 0.0008 | <0.0003 | 0.0007 | 0.0003 | 0.0012 | 0.0025 | 0.0032 | 0.0014 | 0.0008 | <0.015 |
| Se | <0.01 | <0.01 | <0.01 | <0.01 | 0.01 | <0.01 | <0.01 | <0.01 | <0.01 | <0.50 |
| Sb | <0.0002 | <0.0002 | 0.0013 | 0.0004 | 0.0016 | <0.0002 | <0.0002 | 0.0004 | 0.0004 | <0.001 |
| Al | <0.005 | <0.005 | 0.082 | 0.071 | 0.154 | <0.005 | <0.005 | <0.005 | <0.005 | <0.01 |
| Cr | <0.005 | <0.005 | 0.023 | 0.021 | 0.026 | 0.008 | 0.018 | 0.009 | 0.015 | <0.025 |
| Mo | <0.0003 | <0.0003 | 0.0006 | 0.0004 | 0.0021 | <0.0003 | <0.0003 | 0.001 | 0.001 | <0.0015 |
| V | <0.001 | 0.017 | 0.002 | 0.004 | 0.004 | 0.015 | 0.019 | 0.002 | 0.004 | <0.05 |
| Zr | <0.002 | <0.002 | 0.004 | 0.003 | 0.005 | <0.002 | 0.002 | <0.002 | 0.005 | <0.01 |
| Ti | <0.02 | <0.02 | <0.02 | <0.02 | <0.02 | <0.02 | <0.02 | <0.02 | <0.02 | na |
| As | <0.001 | <0.001 | 0.003 | <0.001 | 0.005 | <0.001 | <0.001 | 0.007 | 0.009 | <0.05 |
| Tl | <0.0001 | <0.0001 | 0.0001 | <0.0001 | 0.0002 | <0.0001 | <0.0001 | <0.0001 | <0.0001 | <0.005 |
| W | <0.0003 | <0.0003 | <0.0003 | <0.0003 | <0.0003 | <0.0003 | <0.0003 | <0.0003 | <0.0003 | <0.0015 |
| Cl^-^ | 25596 | 31422 | 25817 | 81593 | 93398 | 11556 | 40203 | 13254 | 84355 | 60228 |
| SO_4_ | <3 | <3 | 3.02 | 3.94 | 3.22 | 747.5 | 2677 | 4.71 | 10.55 | 348.9 |
| HCO_3_ | 167.0 | 89.3 | 839.4 | 190.3 | 119.6 | 403.1 | 308.1 | 302.6 | 78.1 | 108.4 |
| CO_3_ | <0.5 | <0.5 | <0.5 | <0.5 | <0.5 | <0.5 | <0.5 | <0.5 | <0.5 | <0.5 |
| PO_4_ | 0.82 | 0.879 | 1.169 | 0.937 | 1.222 | 0.697 | 0.842 | 0.906 | 0.498 | 1.315 |
| Br | 167.5 | 198.7 | 96.7 | 110.0 | 119.3 | 46.1 | 185.6 | 17.7 | 90.5 | 362.9 |
| I | 198.2 | 156.4 | 3.73 | 41.26 | 198.3 | 5.99 | 22.39 | 2.94 | 11.50 | 25.3 |
| B | 8.24 | 10.23 | 17.88 | 7.82 | 8.01 | 8.46 | 13.19 | 39.67 | 205 | 7.9 |
| Si | 5.86 | 6.41 | 2.58 | 4.06 | 3.78 | 2.31 | 4.83 | 2.98 | 1.95 | 10.07 |
| H_2_S | <0.1 | <0.1 | na | na | na | <0.1 | <0.1 | na | na | na |
| PCC | Na-Cl | Na-Cl | Na-Cl | Na-Cl | Na-Cl | Na-Cl | Na-Cl | Na-Cl | Na-Cl | Na-Ca-Cl |

**References**

Aitchison, J. (1982) The statistical analysis of compositional data. *Journal of the Royal Statistical Society: Series B (Methodological)* **44**: 139–160.

Buccianti, A. and Pawlowsky-Glahn, V. (2005) New perspectives on water chemistry and compositional data analysis. *Mathematical Geology* **37**: 703–727.

Felter, A., Filippovits, E., Gryszkiewicz, I., Lasek-Woroszkiewicz, D., Skrzypczyk, L., Socha, M., et al. (2021) Mapa zagospodarowania wód podziemnych zaliczonych do kopalin w Polsce wg stanu na 31.12.2020 r. Tekst objaśniający do mapy w skali 1:1 000 000, Państwowy Instytut Geologiczny, Warszawa.

Foster, Z.S.L., Sharpton, T.J., and Grünwald, N.J. (2017) Metacoder: an R package for visualization and manipulation of community taxonomic diversity data. *PLOS Computational Biology* **13**: e1005404.

Greenacre, M. (2018) Compositional data analysis in practice, New York: Chapman and Hall/CRC.

Greenacre, M. (2019) Variable selection in compositional data analysis using pairwise logratios. *Mathematical Geosciences* **51**: 649–682.

Greenacre, M. and Lewi, P. (2009) Distributional equivalence and subcompositional coherence in the analysis of compositional data, contingency tables and ratio-scale measurements. *Journal of Classification* **26**: 29–54.

Hron, K., Engle, M., Filzmoser, P., and Fišerová, E. (2021) Weighted symmetric pivot coordinates for compositional data with geochemical applications. *Mathematical Geosciences* **53**: 655–674.

ISO (1989) ISO 9297:1989 — Water quality — Determination of chloride — Silver nitrate titration with chromate indicator (Mohr’s method).

ISO (1994) ISO 9963-1:1994 — Water quality — Determination of alkalinity — Part 1: Determination of total and composite alkalinity.

ISO (2007) ISO 11885 — Water quality — Determination of selected elements by inductively coupled plasma optical emission spectrometry (ICP-OES).

ISO (2016) ISO 17294-2 — Water quality — Application of inductively coupled plasma mass spectrometry (ICP-MS) — Part 2: Determination of selected elements including uranium isotopes.

Krawczyk, J., Mateńko, T., Mądry, J., and Porwisz, B. (1999) Wody lecznicze Buska Zdroju w świetle dotychczasowych badań. *Współczesne Problemy Hydrogeologii* **9**: 159–164.

Kynčlová, P., Hron, K., and Filzmoser, P. (2017) Correlation between compositional parts based on symmetric balances. *Mathematical Geosciences* **49**: 777–796.

Liu, C., Cui, Y., Li, X., and Yao, M. (2021) microeco: an R package for data mining in microbial community ecology. *FEMS Microbiology Ecology* **97**: fiaa255.

Louca, S., Parfrey, L.W., and Doebeli, M. (2016) Decoupling function and taxonomy in the global ocean microbiome. *Science* **353**: 1272–1277.

Lozupone, C. and Knight, R. (2005) UniFrac: a new phylogenetic method for comparing microbial communities. *Applied and Environmental Microbiology* **71**: 8228–8235.

Malinowski, J. ed. (1991) Budowa geologiczna Polski. VII - Hydrogeologia, Wydawnictwo Geologiczne, Warszawa.

McMurdie, P.J. and Holmes, S. (2013) phyloseq: an R package for reproducible interactive analysis and graphics of microbiome census data. *PLOS ONE* **8**: e61217.

Oksanen, J., Simpson, G., Blanchet, F.G., Kindt, R., Legendre, P., Minchin, P., et al. (2022) vegan community ecology package version 2.6-2 April 2022.

Oszczypko, N. and Zuber, A. (2002) Geological and isotopic evidence of diagenetic waters in the Polish Flysch Carpathians. *Geologica Carpathica* **53**: 257–268.

Paczyński, B. and Sadurski, A. eds. (2007a) Hydrogeologia regionalna Polski. I - Wody słodkie, Państwowy Instytut Geologiczny, Warszawa.

Paczyński, B. and Sadurski, A. eds. (2007b) Hydrogeologia regionalna Polski. II - Wody mineralne, lecznicze i termalne oraz kopalniane, Państwowy Instytut Geologiczny, Warszawa.

Paliy, O. and Shankar, V. (2016) Application of multivariate statistical techniques in microbial ecology. *Molecular Ecology* **25**: 1032–1057.

Pluta, I. and Zuber, A. (1995) Origin of brines in the Upper Silesian Coal Basin (Poland) inferred from stable isotope and chemical data. *Applied Geochemistry* **10**: 447–460.

R Core Team (2023) R: a language and environment for statistical computing. *R Foundation for Statistical Computing, Vienna, Austria*.

Rajchel, L. (2016) Źródła solanek z Tyrawy Solnej w płaszczowinie skolskiej Karpat zewnętrznych. *Biuletyn Państwowego Instytutu Geologicznego* **466**: 253–260.

Rajchel, L., Zuber, A., Duliński, M., and Rajchel, J. (2004) Występowanie i geneza wód chlorkowych Soli. *Przegląd Geologiczny* **52**: 1179–1186.

Różkowski, A. (1994) Factors controlling the groundwater conditions of the Carboniferous strata in the Upper Silesian Coal Basin, Poland. *Annales Societatis Geologorum Poloniae* **64**: 53–66.

Różkowski, A. and Przewłocki, K. (1987) The origin of groundwaters in the Upper Silesian Coal Basin (Poland). In *Hydrogeology of coal basins*. Katowice, Poland, pp. 155–170.

Szymakowska, F. (1960) Stratygrafia i tektonika obszaru Tyrawy Solnej - Witryłowa w Karpatach Sanockich. *Biuletyn Państwowego Instytutu Geologicznego* **141**: 237–308.

Tatarski, A. and Iwanowski, S. (1976) Hydrogeological documentation of therapeutic water resources from wells “Tadeusz” and “Korona” in Zabłocie, Unpublished, Solanka z Zabłocia Ltd., Zabłocie.

Templ, M., Hron, K., and Filzmoser, P. (2011) robCompositions: An R-package for Robust Statistical Analysis of Compositional Data. In *Compositional Data Analysis*. John Wiley & Sons, Ltd, pp. 341–355.

Ter Braak, C.J.F. and Prentice, I.C. (1988) A Theory of Gradient Analysis. In *Advances in Ecological Research*. Begon, M., Fitter, A.H., Ford, E.D., and Macfadyen, A. (eds). Academic Press, pp. 271–317.

Wei, T. and Simko, V. (2021) R package “corrplot”: Visualization of a correlation matrix (Version 0.92).

Wei, W. and Koslicki, D. (2022) WGSUniFrac: applying UniFrac metric to whole genome shotgun data. *Leibniz International Proceedings in Informatics* **242**: 15:1-15:22.

Wickham, H. (2016) ggplot2: Elegant Graphics for Data Analysis, Springer International Publishing.

Zuber, A. and Grabczak, J. (1985) Pochodzenie niektórych wód mineralnych Polski południowej w świetle dotychczasowych badań izotopowych. *Aktualne problemy hydrogeologii* 135–148.

Zuber, A., Weise, S.M., Osenbriick, K., and Mateńko, T. (1997) Origin and age of saline waters in Busko Spa (Southern Poland) determined by isotope, noble gas and hydrochemical methods: evidence of interglacial and pre-Quaternary warm climate recharges. *Applied Geochemistry* **12**: 643–660.
